# Energy Landscape Reveals the Underlying Mechanism of Cancer‐Adipose Conversion in Gene Network Models

**DOI:** 10.1002/advs.202404854

**Published:** 2024-09-11

**Authors:** Zihao Chen, Jia Lu, Xing‐Ming Zhao, Haiyang Yu, Chunhe Li

**Affiliations:** ^1^ Shanghai Center for Mathematical Sciences Fudan University Shanghai 200433 China; ^2^ Institute of Science and Technology for Brain‐Inspired Intelligence Fudan University Shanghai 200433 China; ^3^ State Key Laboratory of Component‐based Chinese Medicine Tianjin University of Traditional Chinese Medicine Tianjin 301617 China; ^4^ Haihe Laboratory of Traditional Chinese Medicine Tianjin 301617 China; ^5^ School of Mathematical Sciences and MOE Frontiers Center for Brain Science Fudan University Shanghai 200433 China

**Keywords:** cancer‐adipose conversion, combination drugs, energy landscape, gene regulatory network, mathematical model

## Abstract

Cancer is a systemic heterogeneous disease involving complex molecular networks. Tumor formation involves an epithelial‐mesenchymal transition (EMT), which promotes both metastasis and plasticity of cancer cells. Recent experiments have proposed that cancer cells can be transformed into adipocytes via a combination of drugs. However, the underlying mechanisms for how these drugs work, from a molecular network perspective, remain elusive. To reveal the mechanism of cancer‐adipose conversion (CAC), this study adopts a systems biology approach by combing mathematical modeling and molecular experiments, based on underlying molecular regulatory networks. Four types of attractors are identified, corresponding to epithelial (E), mesenchymal (M), adipose (A) and partial/intermediate EMT (P) cell states on the CAC landscape. Landscape and transition path results illustrate that intermediate states play critical roles in the cancer to adipose transition. Through a landscape control approach, two new therapeutic strategies for drug combinations are identified, that promote CAC. These predictions are verified by molecular experiments in different cell lines. The combined computational and experimental approach provides a powerful tool to explore molecular mechanisms for cell fate transitions in cancer networks. The results reveal underlying mechanisms of intermediate cell states that govern the CAC, and identified new potential drug combinations to induce cancer adipogenesis.

## Graphical abstract

Understanding the mechanism of cancer‐adipose conversion (CAC) is a critical and complex challenge in cancer treatment. A systems biology approach combining molecular network modeling and experiments is proposed based on energy landscape theory, which provides an important way to identify optimal drug combinations driving CAC. This study reveals new therapeutic strategies and provides valuable insights into the mechanisms underlying CAC.

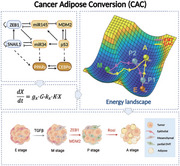

## 1 Introduction

Cancer formation is a complex process with multiple transition states governed by underlying gene regulatory networks.^[^ ^1^, ^2^ ^]^ Tumor cells undergo both epithelial‐mesenchymal transition (EMT) and mesenchymal‐epithelial transition (MET), to achieve plasticity and metastasis.^[^ ^3^, ^4^, ^5^, ^6^ ^]^ The EMT process enhances the plasticity of cancer cells, and is believed to facilitate cancer metastasis.^[^ ^7^, ^8^, ^9^ ^]^ It has been proposed that cells undergoing EMT and/or MET enter a state of heightened plasticity, potentially providing a critical window for therapeutic intervention.^[^ ^7^, ^10^, ^11^ ^]^ For example, recent work suggested that cancer cells can be transformed into adipocytes through a specific combination of treatments.^[^ ^12^, ^13^ ^]^ However, the underlying mechanisms driving this cancer‐adipose conversion (CAC) are unknown at the present time. This motivated us to explore the underlying molecular regulatory networks that govern cell fate decisions in CAC.

Dynamical modeling approaches provide effective tools to analyze the functions and behaviors of biological networks, e.g., for EMT and cancer networks.^[^ ^14^, ^15^, ^16^, ^17^, ^18^ ^]^ Meanwhile, a stochastic description needs to be considered in cell fate decisions, owing to intrinsic fluctuations within cells and external fluctuations acting upon cells.^[^ ^19^, ^20^ ^]^ Energy landscape theory, which builds upon the classic Waddington epigenetic landscape metaphor,^[^ ^21^ ^]^ has been developed to study stochastic dynamics of gene regulatory networks,^[^ ^22^, ^23^, ^24^, ^25^, ^26^, ^27^, ^28^, ^29^, ^30^, ^31^ ^]^ which occurs, for example, in development and cancerization.^[^ ^24^, ^32^, ^33^, ^34^, ^35^ ^]^ From the landscape view, cell types can be characterized by basins of attraction on the landscape, which reflect the probability of appearance of different cell types. States with lower potential (or higher probability) represent attractors or biologically functional states, and constitute the basin of attraction or stable state. From this viewpoint, biological processes like tumorigenesis or differentiation can be understood as transitions from an attractor state to another state in the gene expression state space of the underlying gene regulatory network.^[^ ^32^ ^]^ Moreover, dynamical transition paths, between attractors or cell types, can be quantified from the landscape through minimum action path methods. These approaches yield insights into the sequence of gene activations or deactivations during the transition process, and serve as powerful conceptual/theoretical tools with which to explore the mechanisms of the transition of cells from cancerous to becoming adipose.^[^ ^34^, ^36^ ^]^

In this study, we unravel the mechanisms of the CAC through application of landscape theory. We first construct a gene network model involving the EMT and the adipogenesis regulatory network. Based on a CAC model, we quantify the energy landscape to study the stochastic dynamics of this process.^[^ ^22^ ^]^ We identify four types of attractors on the landscape, which characterize epithelial tumor state (E), mesenchymal tumor state (M), adipose state (A) and two partial/intermediate EMT states (P1 and P2), individually. To quantify the transition process in a CAC, we calculate the kinetic transition path for each transition. Based on the transition path results, we propose that the CAC can be interpreted as a transition from an E or M tumor cell state to an A cell state, which agrees well with bulk RNA‐seq results.^[^ ^12^ ^]^ To explore the underlying molecular mechanism of a drug induced CAC,^[^ ^12^ ^]^ we examine different drug combinations in a CAC gene network model. We find that TGF‐*β* facilitates the M state, MEKi promotes the generation of P states with a certain level of TGF‐*β*, and Rosiglitazone promotes the A state with TGF‐*β* and MEKi. These results support the hypothesis that malignant tumor cells have the potential of passing through a partial EMT state and becoming adipocytes.^[^ ^37^ ^]^ More importantly, the landscape results provide possible theoretical explanations for the mechanism of a CAC through intermediate cell states, from a molecular regulatory network perspective.

To infer other drug combinations, that could induce a CAC, using a landscape model, we focused on pinpointing critical molecular regulatory elements of the CAC. By adopting a landscape control strategy, grounded in the CAC gene network model, we identify two optimal drug combinations. One is a combination of a ZEB1 activator with Rosiglitazone, and the other combines an MDM2 activator with Rosiglitazone.

To validate our predictions, regarding the efficacy of the proposed drug combinations, we conducted experiments using liver, breast, and colon cancer cell lines. We applied oil red staining to these cells to detect lipid droplet accumulation, which is indicative of adipocyte differentiation. Immunofluorescence staining and BODIPY detection, through flow cytometry, revealed a significant increase in lipid droplets within the tumor cells, following treatment with a ZEB1 activator and Rosiglitazone. Furthermore, Western Blot analysis confirmed elevated levels of adipocyte‐specific proteins, while RT‐PCR assays showed an increase in mRNA levels of adipocyte‐related genes after the treatment with a combination drug. Additional RNA‐seq data analyses show that the cancer cells that are treated with a combination of drugs are transformed into cells with more similarities to those of mature adipocytes. These experiments verified our model predictions and therefore provide support that a ZEB1 activator and Rosiglitazone may be an effective combination of drugs for inducing the transition of metastatic tumor cells into adipose cells.

Our findings offer a comprehensive and quantitative perspective on cancer‐adipose conversion (CAC), enhancing the mechanistic understanding of this transition process through insights from the underlying molecular regulatory networks. By integrating computational models with molecular experiments, we have established a robust framework for examining the stochasticity and dynamics of cell fate decisions in tumor cells, thereby laying the groundwork for innovative approaches to cancer treatment.

## 2 Results

### 2.1 Construction of a Cancer‐Adipose Conversion Gene Network Model

**Figure 1:**
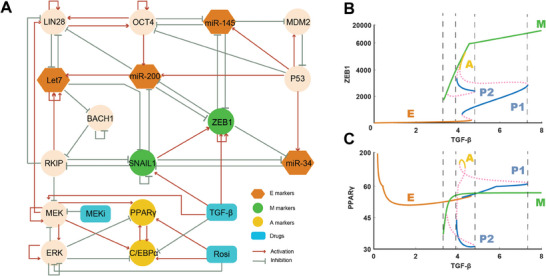
Deterministic dynamical model for cancer‐adipose conversion network. A) Wiring diagram of cancer‐adipose conversion model. The red arrows represent activation and the green bars represent inhibition. Circle nodes represent transcription factors, with green nodes for mesenchymal markers, yellow nodes for adipogenesis markers. Orange hexagonal nodes represent microRNAs, and blue quadrilateral nodes represent drugs, including TGF‐*β*, MEK inhibitor and Rosiglitazone. B) Bifurcation analysis of TGF‐*β* with respect to ZEB1. The solid lines with different colors represent the stable states and the pink dotted lines represent unstable states. E represents the epithelial tumor cell state, M represents the mesenchymal tumor cell state, and A represents the adipose cell state. P1 and P2 represent the partial EMT (intermediate) states. (C) Bifurcation analysis of TGF‐*β* with respect to PPAR*γ*.

To explore the molecular mechanism of the CAC process, we first built a gene regulatory network model by combining EMT and adipogenesis circuits (**Figure** **1**A; Table S1, Supporting Information). Of note, our goal here is not to identify a comprehensive network for the CAC, but to establish a core molecular network that can potentially explain diverse cell fate transitions that are observed in experiments. In fact, recent work suggests that, although regulatory network models may not always include all the regulators involved in cell fate regulations, they still provide incredibly effective tools for understanding cell fate transitions and for making useful predictions.^[^ ^38^ ^]^ To this end, we focus on important markers of the EMT network, based on previous models,^[^ ^14^ ^]^ in which the EMT can be understood as a switching process that is governed by reciprocal inhibition between P53‐induced microRNAs (miR145, miR200, and miR34) and EMT transcription factors (SNAIL1 and ZEB1).^[^ ^7^, ^39^, ^40^ ^]^ For simplicity, we pick ZEB1 and SNAIL1 to represent the ZEB family and the SNAIL family, respectively. Moreover, P53 activates its inhibitor, MDM2, thereby creating a negative feedback loop.^[^ ^41^ ^]^ The cancer metastasis process involves complex regulatory interactions among P53, microRNAs, OCT4, Let7, LIN28, and BACH1.^[^ ^42^, ^43^, ^44^, ^45^, ^46^ ^]^ Specifically, in breast cancer cells, BACH1 facilitates metastasis and suppresses the transcription of the RKIP. Conversely, RKIP promotes Let7 activity and inhibits both LIN28 and the MAPK signaling pathway.^[^ ^18^, ^47^, ^48^, ^49^ ^]^

To acquire a core molecular network of the CAC, we expanded the initial EMT network by incorporating MAPK signaling pathways (highlighted by MEK and ERK), the adipogenesis pathway (marked by PPAR*γ* and C/EBP*α*), and relevant drugs (TGF‐*β*, MEKi and Rosiglitazone).^[^ ^18^, ^37^, ^50^, ^51^ ^]^ Our model captures key genes that are pivotal to both EMT and adipogenesis—such as MEK, ERK, RKIP, SNAIL1, ZEB1, Let7, PPAR*γ*, C/EBP*α*—and their regulatory interactions that play a crucial role in the CAC (Figure 1A). In this network, red arrows represent activation, and green bars represent inhibition (Figure 1A).

With the network structure, to describe the temporal evolution of different components underlying a CAC, we can write down an ordinary differential equation (ODE) model (see Experimental Section and Supporting Information for details, see Tables S2 and S3 (Supporting Information) for model parameters, and Table S3 (Supporting Information) for the robustness analysis of models). The solutions to the ODE model reveals multi‐dimensional gene expression profiles over time (Figure S1, Supporting Information). Depending on different initial conditions, the expression levels of the genes, that are involved, may stabilize at distinct fixed points, referred to as stable states or attractors in the language of dynamical systems. Identifying these attractors within a high‐dimensional dynamical system is crucial, as they correspond to various cell types (Figures S1 and S2, Supporting Information). Moreover, variation in the system's parameters can lead to bifurcations, which change the number of attractors and induce phase transitions. Given that TGF‐*β* and MEKi have been identified as crucial factors for the CAC in prior studies,^[^ ^12^, ^13^ ^]^ we initially conducted a bifurcation analysis of the CAC system, focusing on the effects of TGF‐*β* (Figure 1B,C) and MEKi (Figure S3, Supporting Information).

**Figure 2:**
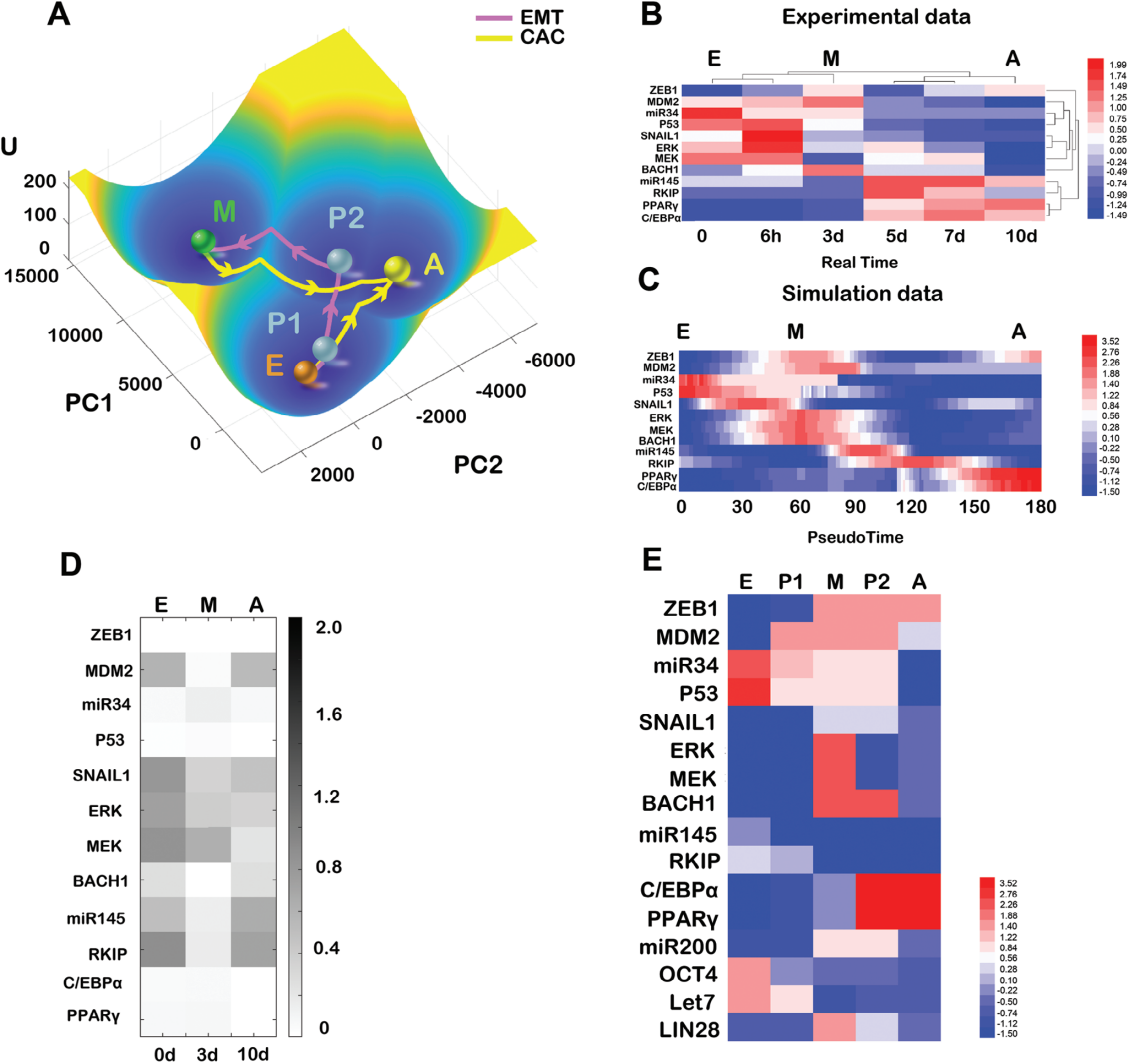
Energy landscape and transition path quantify the transition process for CAC. A) A 3D visualization of the landscape and corresponding transition paths after dimension reduction, focusing on the first and second principal components (PC1 and PC2, derived from gene expression levels within the CAC network as given in Table S3, Supporting Information). Yellow lines indicate transition paths for the CAC, while pink lines map out the EMT process. The U axis represents the dimensionless potential. The transition paths represent the most probable path for each transition, and are obtained by minimizing the transition action. B) Expression levels of different genes in the CAC process, as derived from bulk RNA‐seq data. A clustering method was used to identify the E, M and A phenotypes. By comparing model predictions with experimental data, we infer that the cell state at day 7 aligns with the P2 state. C) Pseudotime series expression profiles of different genes for the transition path from E to M and to A from the model. D) Compares gene expression levels between the model predictions and actual bulk RNA‐seq data, with lighter blocks indicating minor discrepancies. The horizontal axis marks the experimental timeline in days (d). E) Expression patterns for five stable states. Red blocks represent high expression levels, and blue blocks represent low expression levels. E represents Epithelial state, M represents Mesenchymal state, A represents adipose state, and P1/P2 represent intermediate states.

The bifurcation analysis reveals the emergence of multiple attractors in a CAC system, as the concentration of TGF‐*β* steadily increases (Figure 1B,C). This analysis indicates the importance of the expression levels of the mesenchymal marker ZEB1 (Figure 1B) and the adipogenesis marker PPAR*γ* (Figure 1C), and correlates them with varying levels of TGF‐*β*. The analysis identifies up to five stable states (represented by colored solid lines) and three unstable states (indicated by pink dotted lines). By comparing the levels of these stable states with experimental data (**Figure** **2**B), we have classified them as the epithelial tumor state (E), two partial EMT states (P1 and P2), the mesenchymal tumor state (M), and the adipose state (A). At very low TGF‐*β* levels (<3.2), the EMT process does not proceed, and cells remain in the E state. As TGF‐*β* levels increase (3.2 to 3.9), the system supports both E and M states, facilitating an EMT. The attractor landscape becomes more complex with further increases in TGF‐*β*. The P2 state appears between E and M states for TGF‐*β* levels from 3.9 to 4.8. Following the P2 state's emergence, the A state becomes evident as TGF‐*β* reaches levels between 4.1 and 4.4. The P1 state, lying between E and A states, emerges with TGF‐*β* levels spanning 4.3 to 7.3. At relatively high levels of TGF‐*β* (4.9 to 7.3), both M and P1 states coexist. Finally, at very high TGF‐*β* concentrations (>7.5), cells exclusively adopt the M state.

In this study, our primary goal was to facilitate the transition of the system from a cancerous state (either epithelial, E, or mesenchymal, M) to an adipose (A) state. The deterministic dynamic model indicates the presence of multiple partial EMT states during the cancer‐to‐adipose conversion (CAC). To enable the A state's emergence, it is critical to maintain TGF‐*β* at a specific level. Moreover, these partial EMT states act as intermediary phases that aid in the transition from M to A or from E to A. A moderate concentration of TGF‐*β* is essential for cells to undergo transitions from E or M states to A state, navigating through these partial EMT phases (refer to Figure 1B,C).

### 2.2 Energy Landscape and Transition Path Quantify the Transition Process for the Cancer‐To‐Adipose Conversion

A bifurcation analysis offers insight into a system's multistable states from a deterministic perspective. However, it is crucial to account for stochastic effects in the dynamics of the cancer‐to‐adipose conversion process, since intracellular fluctuations can significantly influence cellular behaviors.^[^ ^20^, ^52^, ^53^ ^]^ To disentangle the transition mechanism and study the stochastic dynamics of the CAC, we quantified the energy landscape using the CAC gene network model and have previously developed methodologies for this (see Experimental Section).^[^ ^22^, ^23^, ^24^, ^25^, ^34^ ^]^ For visualization purposes, we employed a dimension reduction technique to represent the landscape in a 2D space (Figure 2A; Table S4, Supporting Information).^[^ ^54^ ^]^ On the landscape the blue region represents lower potential and higher probability while the yellow region represents higher potential and lower probability. This analysis identified five attractors corresponding to the E, M, A, P1, and P2 cell states, with their gene expression levels given in Table S6 (Supporting Information).

To quantify the transition processes among different cell types, characterized by attractors on the landscape, we employed a minimum action paths (MAPs) method to obtain the most probable transition path for each change.^[^ ^55^ ^]^ These MAPs connect the different cell states, with arrows indicating the transitions between attractors. Pink arrows depict the sequence E→P1→P2→M, representing the EMT process. In contrast, yellow arrows show the transitions from either E or M states to the adipose (A) state, passing through either the P1 or P2 intermediate states. The landscape featuring five attractors illustrates how, during an EMT, epithelial tumor cells evolve into mesenchymal tumor cells, while adipogenesis might hijack the EMT toward another committed cell fate, i.e., the adipose cell state.^[^ ^37^ ^]^

Previous studies have documented three distinct states in the CAC, corresponding to the E, M and A states.^[^ ^12^ ^]^ We aligned the RNA sequencing data from mesenchymal breast cells (Figure 2B) with the pseudo‐time expression profiles generated by our model (Figure 2C) to validate the landscape model and its transition paths. Given that our model starts from the E state, while the experimental setup began with the ablation of the E‐Cadherin gene,^[^ ^12^ ^]^ some discrepancies in gene expression at the outset are anticipated (Figure 2B,C). Nevertheless, our simulation data largely agree with the experimental findings, in terms of key gene expressions. For instance, the high expression levels of p53 and miR34 in the E state highlight epithelial characteristics (Figure 2B,C). Both the model and experimental data show similar expression patterns in the M state, with elevated levels of ZEB1 and BACH1 (Figure 2B,C). Regarding the A state, both simulation and experimental data demonstrate high expression of adipogenesis markers PPAR*γ* and C/EBP*α*. (Figure 2B,C). We depicted the variance in expression levels for genes across the E, M, and A states between our model and the experimental data, where lighter blocks indicate minor differences (Figure 2D). This comparison reveals that our model aligns closely with the experimental RNA‐seq data (Figure 2D).^[^ ^12^ ^]^ We also show the expression patterns of the five attractors (fixed points) on the landscape (Figure 2E, corresponding to Table S6, Supporting Information). From the expression pattern of the five stable states, the E state has the highest expression level of P53, while the M state has the highest expression of MEK, ERK and SNAIL1. P1 and P2 states have lower level of MEK and ERK expression than the M state, and less p53 expression than the E state. P2 and A states both have higher expression levels of adipose markers PPAR*γ* and C/EBP*α*.

Drawing from the landscape analysis, we identify two primary pathways leading to the adipose (A) state: one from the epithelial (E) state via the partial EMT state (P1) and another from the mesenchymal (M) state via the partial EMT state (P2) (Figure 2A). Applying a clustering method to RNA sequencing data from mesenchymal breast cells led to the identification of four distinct clusters, corresponding to the E, M, and A states, along with an additional cluster.^[^ ^12^ ^]^ Through gene expression comparison (Figure 2B,C), we speculate that this additional cluster (experimental data for 7 days, Figure 2B) is the P2 intermediate state, which supports our modeling results on the intermediate states. Further support for this interpretation comes from the expression profiles along the multi‐dimensional transition paths, where we pinpointed states resembling P1 and P2 during the transitions from E to A and M to A, respectively, evidenced by Pearson correlation coefficients (Figure S4A–D, Supporting Information). We need to emphasize that, previous work^[^ ^12^ ^]^ discussed the possible roles of intermediate states, but no quantitative results or the evidence of existence of intermediate states in the CAC were presented. Our comprehensive molecular network model not only substantiates the presence of these intermediate states during a CAC but also clarifies their role and dynamics in the process.

### 2.3 MEK Inhibitor and Rosiglitazone Induce the Transition of Cancer Cells into Adipose Cell Through Partial EMT State

**Figure 3:**
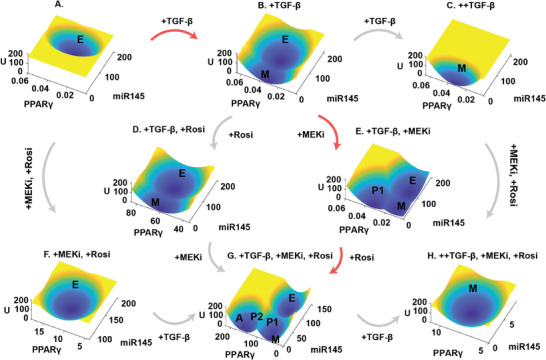
Landscapes recapitulate the effects of drug combinations used in previous experiments for inducing a CAC. A–H) Landscape with different doses and combinations of TGF‐*β*, MEK inhibitor (MEKi) and Rosiglitazone (Rosi). The landscape is projected onto the miR145 axis (characterizing EMT level) and the PPAR*γ* axis (characterizing the level of adipogenesis). The U axis is the dimensionless potential. The red arrows indicate the route for drug additions of inducing the CAC proposed in previous experiments.^[^ ^12^ ^]^ Here we use “+” to mark the level of the drugs. (A) TGF‐*β*, MEKi and Rosiglitazone are not added, and the system has a monostable E state, with TGF‐*β* = 1, MEKi = 0.001, Rosi = 0.1. (B) Moderate TGF‐*β* is added (+TGF‐*β* or TGF‐*β* = 4.3), but MEKi and Rosiglitazone are not added. (C) High level of TGF‐*β* is added (++TGF‐*β* or TGF‐*β* = 8), but MEKi and Rosiglitazone are not added. (D) TGF‐*β* and Rosiglitazone are added (+TGF‐*β*, +Rosi or Rosi = 1.5), but MEKi is not added. (E) TGF‐*β* and MEKi are added (+TGF‐*β*, +MEKi or MEKi = 0.02), but Rosiglitazone is not added. (F) MEKi and Rosiglitazone are added (+MEKi, +Rosi), but TGF‐*β* is not added. (G) Moderate TGF‐*β*, MEKi and Rosiglitazone are added (+TGF‐*β*, +MEKi, +Rosi). (H) High level of TGF‐*β*, MEKi and Rosiglitazone are added (++TGF‐*β*, +MEKi, +Rosi).

To clarify the stochastic mechanism of cell fate commitment in a CAC, we altered the key regulators and tracked the changes of attractors on the landscape (**Figure** **3**). To visualize and analyze the high‐dimensional landscape, we projected the landscape onto a 2D plane to visualize multiple attractors (Figure 3). In this projection, we selected PPAR*γ* (characterizing the level of adipogenesis) and miR145 (characterizing the level of E state) as the two coordinates. These efforts allow us to understand the CAC from the two coordinates of EMT and adipogenesis. Additionally, we presented landscape analyses using alternative gene pairs as coordinates to underscore the robustness of our findings (Figure S6, Supporting Information). We need to emphasize that our results are based on the full gene network (Figure 1A), and the landscape is shown in reduced dimensions for the visualization purpose.

To see how the landscape changes with different drug additions, we start from the E state cells without TGF‐*β* addition (Figure 3A). The E state has a low level of PPAR*γ* but a high level of miR145 (Figure 3A). We then simulated the effects of adding Rosiglitazone and MEKi, but without TGF‐*β* (Figure 3F). This combination elevated PPAR*γ* levels but was insufficient to trigger a phase transition in the absence of TGF‐*β*, leaving the epithelial state unchanged (Figure 3F). In another simulation, we introduced a moderate level of TGF‐*β* (+TGF‐*β*) without MEKi and Rosiglitazone (Figure 3B). In this case, we observed the coexistence of the E and M states (a bistable state), likely due to TGF‐*β*’s role in promoting the EMT process. In this situation, the E state has a higher level of miR145, while E and M states have similar levels of PPAR*γ* (Figure 3B). Increasing TGF‐*β* to higher levels (++TGF‐*β*) shifted all cells to the M state, characterized by reduced miR145 and PPAR*γ* levels (Figure 3C). Of note, the addition of Rosiglitazone and MEKi under high level of TGF‐*β* (++TGF‐*β*) is not able to induce P state or A state (Figure 3H). These results illustrate that an appropriate range of the TGF‐*β* level is required for inducing CAC. That is because at a low level of TGF‐*β* the system is in a monostable E state (Figure 3A,F), while at a high level of TGF‐*β* the system is in a monostable M state (Figure 3C,H). To make the transition to A state possible, we need the system being pushed to a multistable state with A state and intermediate states (Figure 3G). Therefore, the landscape results with different drug additions (Figure 3) explain why TGF‐*β* needs to be in a middle range so that the CAC is possible.

However, the mere addition of TGF‐*β* is insufficient to produce the P1 or A states (Figure 3A–C), indicating that Rosiglitazone and MEKi are essential components for facilitating CAC. To further clarify the roles of MEKi and Rosiglitazone in the CAC, we maintained TGF‐*β* at an optimal level (+TGF‐*β*) and introduced MEKi and Rosiglitazone separately (Figure 3D,E). The introduction of Rosiglitazone, with TGF‐*β* at a moderate level (+TGF‐*β*), preserved the system's bistability, exhibiting both E and M states (Figure 3D). Conversely, adding MEKi at the same level of TGF‐*β* (+TGF‐*β*) resulted in a system with three stable states: E, M, and P1, with P1 positioned between E and M states under the EMT framework and exhibiting higher PPAR*γ* levels than both M and E states (Figure 3E). This demonstrates that MEKi, in conjunction with TGF‐*β*, can facilitate the emergence of the P1 state.

With only a moderate level of TGF‐*β* (+TGF‐*β*) added, the system remained in a bistable state without MEKi and Rosiglitazone (Figure 3B). However, the simultaneous addition of TGF‐*β* at a moderate level (+TGF‐*β*), MEKi, and Rosiglitazone led to a system with five stable states (Figure 3G). The P2 state has higher level of PPAR*γ* than P1 and M states, while A state has a significantly higher level of PPAR*γ* than other states (Figure 3G). These results suggest that Rosiglitazone combined with TGF‐*β* and MEKi promotes the adipogenesis by promoting the P2 state and A state (Figure 3G). MEKi seems to enhance the system's capacity to generate intermediate (or partial EMT) states, while Rosiglitazone aids in transitioning from these partial EMT states to the adipose state (Figure S7, Supporting Information). Interestingly, our model aligns with experimental observations regarding drug interventions, highlighting that the order of administering MEKi and Rosiglitazone does not alter the CAC outcome or landscape^[^ ^12^, ^13^ ^]^ (Figure 3). Thus, the insights from our gene network model corroborate experimental data on drug combinations that induce CAC, providing a quantitative framework for understanding the mechanism behind the effectiveness of these drug combinations in inducing CAC.

### 2.4 Landscape Control Identifies new Drug Combinations for Inducing CAC

**Figure 4:**
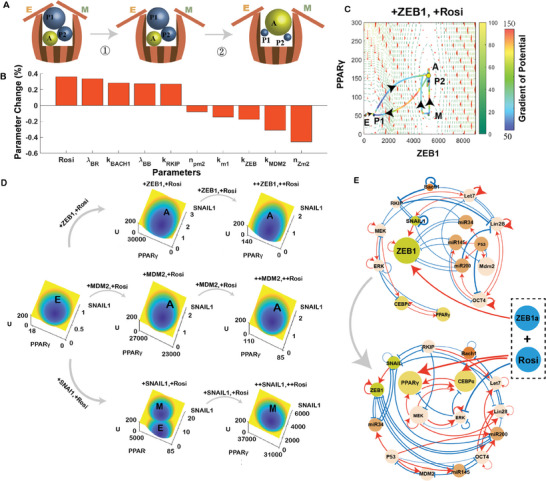
Landscape control identifies new drug combination for inducing CAC. A) Schematic diagram of the CAC process. B) Critical elements to increase the occupancy of A state identified from landscape control. The x axis shows the parameters identified from landscape control with the top 10 absolute values of relative change. The y axis shows the relative change ratio of the parameters identified by landscape control with respect to their initial value. C) Gradient of potential with the addition of the ZEB1 activator and Rosiglitazone, with larger balls corresponding to higher occupancy. The color along the paths represents the gradient of potential. The occupancy of A state is enhanced after addition of the ZEB1 activator and Rosiglitazone. E, epithelial state; M, mesenchymal state; A, Adipose cell state; P, partial EMT (intermediate) state. D) Landscape changes for different drug combinations. Gray arrows represent the direction and order of the dosing, with all simulations beginning with no drug additions. The first group of simulated drugs combines the ZEB activator and Rosiglitazone with drug level of ten times (first row, second column) and 500 times (first row, third column). The second group of simulated drugs combines the MDM2 activator and Rosiglitazone with drug level of 10 and 100 times (second row). The third group of simulated drugs combines the SNAIL1 activator with Rosiglitazone at drug level of 10 and 100 times (third row). E) Key drug combinations for the ZEB1 activator and Rosiglitazone identified from landscape control in terms of CAC network (corresponding to Figure S8A1,A7, Supporting Information). The thickness of the arrows is proportional to the sensitivity of the parameters obtained from the landscape control, and the size of the balls is proportional to the change of the ratio between the synthesis rate and degradation rate for each node.

The landscape with five stable states (Figure 2A) offers a comprehensive perspective on the dynamics of the CAC, depicting it as a transition from the basins representing epithelial (E) or mesenchymal (M) states toward the adipose (A) state basin. The CAC process can be also understood as a two‐step process illustrated by a cartoon (**Figure** **4**A). The first step is to induce the appearance of the A state and intermediate (P1 and P2) states. The second step is to increase the occupancy of the A state. The balls in the bucket represent the P1, P2 and the A state in the EMT process, and the two pieces of wood on the barrel represent the E state and M state. Without the drug treatment, E and M states are the only two stable states and dominate the performance of the system, while the intermediate (P1 and P2) states and the A state are hard to appear (Figure 4A).

Our results resemble the idea that cancer cells can be characterized by attractors,^[^ ^56^ ^]^ which are determined by underlying regulatory networks. So, therapeutically, a good strategy should be to target the cancer network, in order to induce a cell fate transition, from cancer cell attractors to non‐malignant cell attractors.^[^ ^14^ ^]^ Targeting the fat metabolism pathway has been shown to be a promising way against cancer in different tumor types.^[^ ^57^, ^58^, ^59^ ^]^ Several drugs have been suggested to be effective against cancers through MAPK pathway.^[^ ^60^ ^]^ Nevertheless, these drugs focus on killing tumor cells,^[^ ^61^ ^]^ but not changing the landscape of CAC gene networks, such as the stability of E and M states. We reason that traditional strategies for killing tumor cells fail to change the topological structure of the landscape and thus might be ineffective for cancer treatment or lead to cancer relapse.^[^ ^32^ ^]^ We propose that a better way for cancer treatment should be changing landscape topography, i.e., making cancer state less stable, such as inducing transitions from cancer cell states to adipose cell states through targeted perturbations in cancer gene networks. Through single‐factor global sensitivity analysis of model parameters, critical factors influencing the stability of different attractor states can be identified (Figures S11–S13, Supporting Information). Moreover, combination therapies may prove more effective than single‐drug approaches,^[^ ^12^, ^62^ ^]^ as altering a single network node might not sufficiently change the network's landscape. In contrast, targeting multiple genes or regulatory processes simultaneously could achieve more significant landscape modifications (Figure 3). Therefore, a key challenge is identifying optimal drug combinations that can induce the transition from cancer cells to non‐malignant cells, such as adipose cells, effectively altering the cancer treatment paradigm.

To enable adipogenesis of malignant cancer cells as a therapeutic option, our objective is to simplify the treatment approach by identifying a minimal set of drug targets within the CAC network. So, the question becomes how to identify the minimal optimal combinations of drug targets that can induce a cell fate transition from cancer cells to adipose cells, since there will be numerous combinations for drug targets for a large gene network, such as the one we studied here (Figure 1). To address this, we employed a landscape control method, to identify the most important regulations to maximize the occupancy of the A state.^[^ ^8^, ^33^, ^63^ ^]^ The purpose of landscape control is to find a set of parameters which make the objective function achieve the maximum (or minimum) value. Here, we use an objective function based on transition actions, to identify the optimal combinations of targets that can lead to the highest occupancy of the A state (Figure 4B, see Methods for details of landscape control). In the landscape control results, n represents the coefficient of Hill function, and k_I_ represents the generation rate of the transcription factor I. We performed seven experiments with different initial parameter values (Figure S8A,B, Supporting Information) and used the average results as the landscape control results (Figure 4B). We then picked the top ten targets (parameters) according to the absolute values of sensitivity (defined as the percentage change of each parameter from its baseline to the value post‐landscape control adjustment) (Figure 4B). This means that the first five parameters should be increased to promote the A state (predicted to be a positive change from the landscape control), and the latter five parameters should be decreased to promote the A state (predicted to be a negative change from the landscape control) (Figure 4B). Additionally, we propose another potential drug combination to encourage a CAC (refer to Figure S10 and Table S5, Supporting Information) and demonstrate the landscape control method's robustness against variations in initial parameter values, reflecting the heterogeneity of tumor populations (Figure S8, Supporting Information).

Since the hill coefficient n, characterizing the cooperativity for regulations, and regulatory strength (λ) are usually hard to be controlled as a drug target in biological experiments, we pick three major targets from top ten parameters: Rosiglitazone (Rosi), the synthesis rate of ZEB1 (k_ZEB_), and the synthesis rate of MDM2 (k_MDM2_). Therefore, based on the landscape control results (Figure 4B,C; Figure S8, Supporting Information), we identify two potential drug combinations, one is activation of ZEB1 and the addition of Rosiglitazone, another is activation of MDM2 and the addition of Rosiglitazone. As an example, we will focus on the drug combinations of ZEB1 and Rosiglitazone. We increased the synthesis rate of ZEB1 and Rosiglitazone to the optimized level identified from the landscape control (Figure 4C). Here, the size of the ball represents the occupancy of the corresponding attractor states. We find that the occupancy of the A state increases and the occupancy of the M state decreases after adding the combination drugs of ZEB1 and Rosiglitazone. Furthermore, all the transition paths are directed toward to the A state, indicating that this combination of drugs could facilitate the CAC process and enhance the transition toward the A state (Figure 4C). Additionally, the knockdown results of the ZEB1 and Rosiglitazone also validate their significant influence on the CAC (Figure S9, Supporting Information). We further observed that at a relatively low level of ZEB1 generation rate and Rosiglitazone addition, the system shifted to a bistable state system (Figure S9, Supporting Information). As previously mentioned (Figure 4A), the system with only E and M states is unable to generate A state (Figure S9, Supporting Information).

Following the landscape control results, we further tested the effects of two kinds of drug combinations from modeling (Figure 4D). We compared them with a control group consisting of SNAIL1 activation and Rosiglitazone, which was not highlighted in our landscape control analysis (Figure 4D). We quantified the drug effects by perturbing the corresponding targets in the network and tracing the attractor modifications. Initially, all simulations started from a baseline where no TGF‐*β*, MEKi, Rosiglitazone, or other drugs were added, leaving all cells in the epithelial (E) state (Figure 4D). The first drug combination, i.e., adding the activator of ZEB1 and Rosiglitazone can convert the E state to the A state successfully, with tenfold or 500‐fold dose of the drugs (Figure 4D, the first row). The second drug combination, i.e., adding the activator of MDM2 and Rosiglitazone can also transform the cells to the A state (Figure 4D, the second row). The third drug combination, which is the activator of SNAIL1 and Rosiglitazone, was tested as a control example that was not in our prediction (Figure 4D, the third row). SNAIL1 is another important transcription factor in the EMT process. Activation of SNAIL1 and Rosiglitazone with tenfold dose can induce the transition from the E state to the M state (Figure 4D). However, as the concentration of the drug combination was increased to 500‐fold, high levels of the combination caused all of the cells to transition to the M state (Figure 4D), without generating adipose cells. In our network, SNAIL1 directly inhibits the RKIP, which is an inhibitor to the MEK pathway. The activation of SNAIL1 might activate the MEK pathway, which goes against the CAC. This might be why ZEB1 can promote the appearance of the A state, but not for SNAIL1. These results partially support the effectiveness of combination drugs identified from the landscape control.

As shown on the landscape, the drug combinations predicted from landscape control can generate monostable A state (Figure 4D, the first and second row), offering an improvement over previous experimental approaches that combined TGF‐*β*, MEK inhibitor, and Rosiglitazone,^[^ ^12^ ^]^ which will generate multiple cell types including both adipose cell and other tumor cell states (Figure 3, red path). We delve into this result from a network analysis perspective (Figure 4E), where the thickness of the network edges indicates the regulatory influence strength, as determined by the sensitivity of parameters through landscape control. In this network model, the activator of ZEB1 (denoted as ZEB1a) enhances the suppression of P53‐induced microRNAs (Figure 4E), facilitating the EMT process and creating opportunities for intermediate states to emerge. (Figure 4E). From the network perspective, Rosiglitazone activates the adipogenesis markers C/EBP*α* and PPAR*γ* which promote adipogenesis. Notably, prior research highlights that ZEB1 influences a wide array of transcription factors that encourage fat cell development, integrating into the adipogenic gene regulatory network's core.^[^ ^64^ ^]^ These insights offer a clear and quantitative rationale for the effectiveness of the designed drug combinations in triggering CAC.

### 2.5 Molecular Experiments Verified the Effectiveness of Drug Combination for Inducing Cancer Cell Adipogenesis

**Figure 5:**
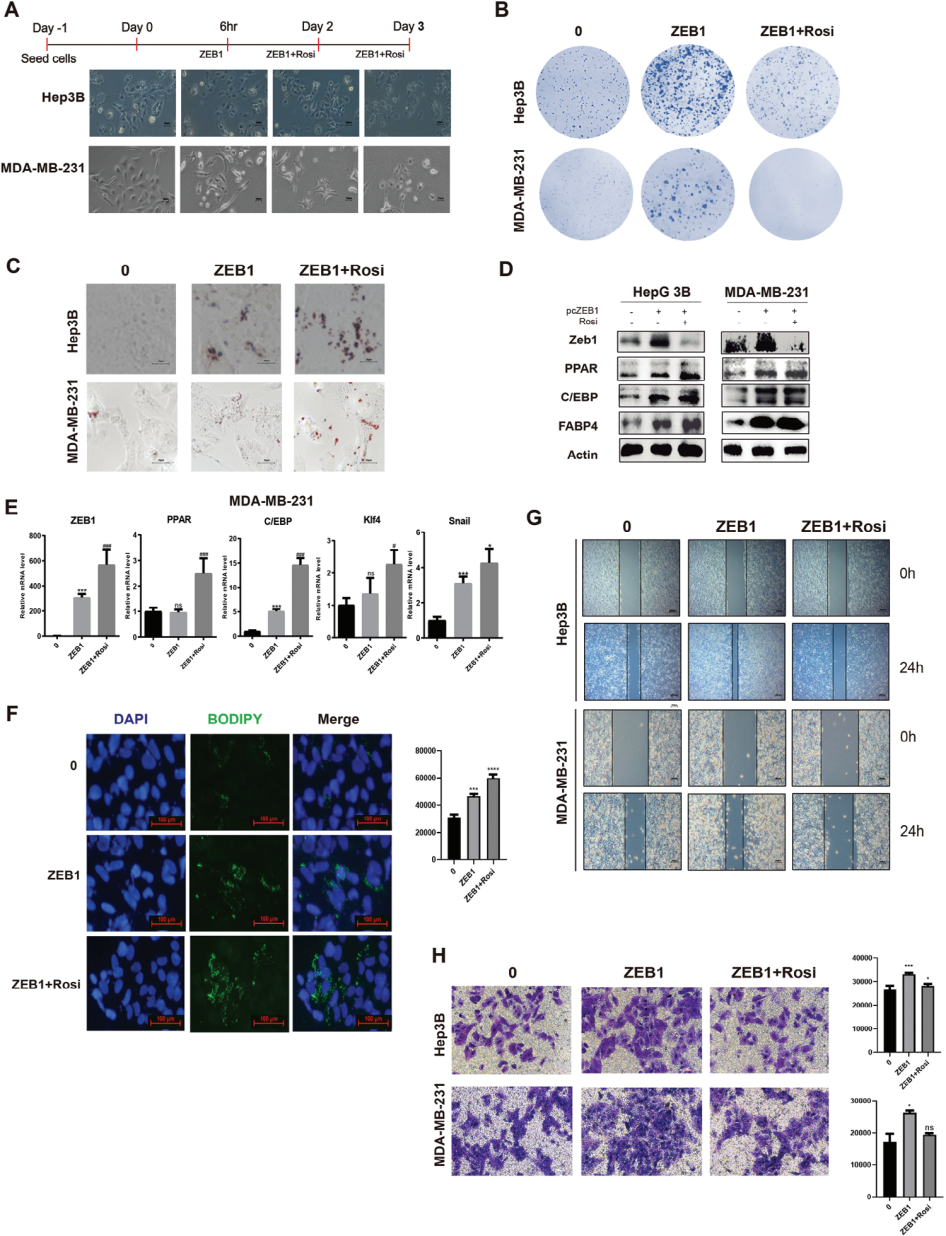
The combination of Rosiglitazone and overexpressing ZEB1 promotes the transformation of tumor cells into adipose cells. A) Morphological changes of Hep3B and MDA‐MB‐231 cells exposed to the ZEB1 activator and Rosiglitazone for the time indicated (magnification, x100). B) Colony‐formation assays show that ZEB1 overexpression combined with Rosiglitazone reduce the proliferation of Hep3B and MDA‐MB‐231 cells. C) Oil red staining was used to detect intracellular lipid droplets in the Hep3B and MDA‐MB‐231 cell lines (magnification, x100). D) The expression of adipocyte‐related proteins was verified by Western Blot. E) Changes in related gene expression (RNA) levels such as ZEB1, PPAR*γ*, C/EPB*α*, Klf4 and Snail were detected by RT‐PCR. F) Bodipy changes in Hep3B cells after ZEB1 overexpression and Rosiglitazone combined treatment were detected by immunofluorescence staining and flow cytometry. G,H) Overexpressing ZEB1 and addition of Rosiglitazone reduce the migration of tumor cells as shown in transwell and Wound‐healing assays.

Our modeling outcomes suggest that a CAC can be facilitated through a drug‐mediated EMT process. Building on this foundation, we conducted molecular experiments to evaluate the efficacy of two drug combinations identified via landscape control for promoting a CAC. For the experimental validation, we chose aggressive cell lines from liver (Hep3B, Huh‐7), breast (MDA‐MB‐231), and colorectal (SW480, SW620) cancers to create ZEB1 overexpression models. Upon overexpression of ZEB1, cells exhibited increased volume and proliferation rates. Subsequent treatment with Rosiglitazone for 24 h induced morphological changes toward a more circular shape (**Figure** **5**A). Colony formation assays in Hep3B and MDA‐MB‐231 cell lines indicated that ZEB1 overexpression spurred cell proliferation, which was inhibited by Rosiglitazone treatment (Figure 5B). To assess lipid droplet accumulation, we employed oil red staining, observing enhanced lipid droplet formation with combined ZEB1 overexpression and Rosiglitazone treatment compared to ZEB1 overexpression alone (Figure 5C).

Lipid metabolism and adipogenesis markers, including PPAR*γ*, C/EBP*α*, and Fabp4, were analyzed through western blot and qRT‐PCR, revealing that ZEB1 overexpression alongside Rosiglitazone significantly boosts lipid metabolism and adipogenesis (Figure 5D). Similar outcomes were noted in liver (Huh‐7) and colorectal (SW480, SW620) cancer cells (Figure S16A,B, Supporting Information). BODIPY lipid staining further confirmed an increase in lipid droplets following the combination treatment (Figure 5F). Additionally, to explore the effects on tumor cell invasiveness, wound healing and transwell assays were performed. Results demonstrated that while ZEB1 overexpression alone heightened tumor invasiveness, subsequent Rosiglitazone treatment markedly reduced this effect, impacting tumor cell migration (Figure 5G,H). Notably, the breast cancer cell line (MDA‐MB‐231) we used here was a P53 mutant cell line, and the liver cancer cell line (Hep3B) was a P53 wildtype cell line. Thus, our combination drugs worked for both cell lines with and without P53 mutation, which was also supported by our modeling results (Figure S14, Supporting Information).

We further tested the effects of another drug combination from our model prediction, i.e., the addition of Rosiglitazone and overexpression of MDM2. Notably, in the Hep3B cell line (Figure S16C, Supporting Information), MDM2 protein levels were found to increase with ZEB1 overexpression, likely due to ZEB1's indirect activation of MDM2 mediated by miR‐145 (Figure 1A). The effects of combining MDM2 overexpression with Rosiglitazone on facilitating CAC were further confirmed experimentally. As shown in Figure S16D,E (Supporting Information), immunofluorescence positive staining for BODIPY and flow cytometry for BODIPY showed that with the combined treatment the tumor cell lipid droplets were increased. At the same time, transwell assay showed that tumor invasiveness was enhanced after overexpression of MDM2, and the addition of Rosiglitazone significantly reduced the ability of the tumor cell migration (Figure S16F, Supporting Information).

To further test the effects of combination drugs (ZEB1 activator and Rosiglitazone) on inducing a cancer to adipose transition, we performed bioinformatics analysis to the RNA‐seq data from our experiments (Supporting Information, Supplemental methods). The results are shown in Figure S17 (Supporting Information) (MDA‐MB‐231 cell line and Hep3B cell line). For MDA‐MB‐231 cell line, the ZEB1 overexpression group contains 7198 differentially expressed genes compared to the untreated group, and the group with overexpression of ZEB1 and Rosiglitazone contains 4184 differentially expressed genes compared with the ZEB1 overexpression group (Figure S17A, Supporting Information, top panel). Differentially expressed genes are significantly reduced in the Hep3B cell line (Figure S17A, Supporting Information, bottom panel). We used the KEGG database to further identify the enriched pathways with differential gene expression (FDR < 0.1, p value < 0.05). These results show that when comparing the untreated group and the group with overexpression of ZEB1 and Rosiglitazone, differential genes are mainly enriched in TNF signaling pathway, p53 signaling pathway, cell cycle‐related pathways, and MAPK signaling pathway (Figure S17B, Supporting Information, left panel), as well as apoptosis, glycolysis, and metabolism related pathways (Figure S17B, Supporting Information, right panel). These pathways are all critically related to the hallmarks of cancer,^[^ ^1^, ^2^ ^]^ illustrating the potential of combination drugs inducing the cell fate transition of tumor cells. We further select several typical pathways (large gene number and small p‐value) for the heat map analysis. The results show that genes related to cell migration ability, such as SERPINE1, are significantly downregulated in the group with combination drug treatment, while the cell cycle inhibitory genes, CDKN1A, TP53, and PMAIP1, are significantly upregulated in the group with combination drug treatment (Figure S17C). CEBP*α*, which plays a significant role in adipogenesis, is also upregulated after treated with ZEB1 overexpression and Rosiglitazone. These results support that the treated cells with combination drugs become less aggressive.

To further investigate whether the induced cells are similar to adipose cells, we explored the correlation between different groups after combination drug treatment and the human Simpson‐Golabi‐Behmel syndrome (SGBS) preadipocyte cell, a typical tool for studies of human adipocytes (GSE161111).^[^ ^65^ ^]^ Correlation analyses reveal a close resemblance of the differentiated states in SGBS cell line adipogenesis (day 7) with our induced cells after combination drug treatment (correlation coefficient increases from 0.69 to 0.72 for MDA‐MB‐231 cell line, and correlation coefficient increases from 0.52 to 0.74 for Hep3B cell line, compared with untreated cells, Figure S17D, Supporting Information), which illustrates that the cancer cells with combination drug treatment are induced to cells with more characteristics of mature adipocytes. In summary, the RNA‐seq results further support that our predicted combination drugs are effective for inducing a transition from cancer cells to adipose cells.

## 3 Discussion

A critical hallmark of an EMT in metastasis formation is the acquisition of “cancer stem cell” properties, which leads to the resistance to therapy.^[^ ^66^, ^67^, ^68^, ^69^ ^]^ Traditional cancer drugs which focus on their killing power against tumor cells might be troubled with EMT.^[^ ^70^ ^]^ So, inducing the conversion of cancer cells into adipocytes, which benefits from the EMT, might be a better way in cancer treatment. Previous studies proposed that the combinations of TGF‐*β*, MEKi and Rosiglitazone will contribute to the CAC, while the underlying mechanisms have yet to be further clarified.^[^ ^12^, ^13^ ^]^

In this study, we constructed a comprehensive gene regulatory network model characterizing the transition process from cancer to adipose cells. To provide a holistic view of the stochasticity and dynamics in the CAC process, we quantified the landscape and transition paths among the five attractors, which correspond to the E, A, M states and two intermediate states (P1 and P2). Some studies reported that there were more than one intermediate states during EMT,^[^ ^15^, ^71^, ^72^ ^]^ while our results revealed the crucial role of the intermediate states in a CAC. Previous experiments observed the transition from the M state to the A state,^[^ ^12^, ^13^ ^]^ while our landscape results further revealed two possible transitions, including E→P1→A and M→P2→A (Figure 2A). This was supported by the pseudotime series expression profiles, which matched the bulk RNA‐seq data in the number of clusters and the expression level of regulatory genes. Thus, our analysis clarifies the dynamical mechanism for the transition of cancer cells into adipose cells through the partial EMT (intermediate) states.

To identify the optimized drug combinations for inducing CAC, we employed a landscape control approach, and revealed the key factors to promote the A state. We propose two new drug combinations for promoting CAC, including raising the ZEB1 and Rosiglitazone, and raising the MDM2 and Rosiglitazone. Our molecular experiments further verified the effects of the two combination drugs on promoting CAC (Figure 5; Figure S16, Supporting Information).

Previous work suggested a drug combination including TGF‐*β*, MEK inhibitor, and Rosiglitazone for inducing CAC.^[^ ^12^, ^13^ ^]^ Here, the drug combinations we identified are simpler than theirs in terms of potential for clinical applications, since we only need two drugs for inducing CAC. From modeling results, we also found that the previous combinations with TGF‐*β*, MEK inhibitor, and Rosiglitazone focused on generation of the A state, which failed to make all the stable states associated with tumors become unstable (Figure 3), while our results provide optimized drug combinations to promote CAC, by eliminating all the tumor associated states and only keep the stable A state (Figure 4E). Also, our molecular network models revealed the underlying molecular mechanism for CAC regarding the critical roles of intermediate states.

An important insight from the landscape of the CAC is that in the process of transformation from E or M state to A state, cells go through multiple intermediate states. Recent studies have identified intermediate hybrid phenotypes both at single‐cell level and population level across different cancer types.^[^ ^7^, ^72^, ^73^ ^]^ How P1 and P2 state can be detected explicitly in molecular experiments warrants further explorations. It is important to note that cancer is a complex disease, which involves many hallmarks,^[^ ^1^, ^2^ ^]^ and this work only modeled a few of these hallmarks (including EMT, metastasis and adipogenesis) using a core molecular network of CAC. Future work can incorporate other critical genes or circuits (and/or other hallmarks of cancer), e.g., important EMT factors TWIST, SNAIL2 and ZEB2, into the models of cancer regulatory networks,^[^ ^14^, ^74^ ^]^ which may provide more insights into underlying regulatory mechanisms for cancer metastasis and CAC. Our modeling framework also allows for integrating multiple feedback loops between distinct genes or pathways, given the network structure. For example, by integrating the autoregulation of TGF‐*β* into the CAC network, we can obtain consistent results for multiple stable states (Figure S15, Supporting Information).

In this work, our major conclusions focus on the fate transition from cancer cells to adipose cells. We investigate this problem by focusing on the EMT circuit and adipogenesis circuit as well as their interactions. Our experiments have successfully verified the effects of the combination drugs for inducing CAC. However, the tumor metastasis is a very complicated process which involves other molecular regulatory networks. How to regulate the metastasis ability of these adipose cells (e.g., control the dose of drugs) warrants further explorations from both theoretical and experimental efforts.

In summary, our work provides a comprehensive understanding of cancer‐adipose conversion through gene network modeling. The landscape and transition paths offer a framework for understanding the underlying mechanisms of cell fate decisions in cancer network and help to design principles to optimize the combination drug strategies for cancer treatment.

## 4 Experimental Section

### Cancer‐Adipose Conversion Model

Previous work regarding the regulatory circuits of EMT and adipogenesis^[^ ^8^, ^41^, ^44^, ^46^, ^75^ ^]^ was summarized, and an ODE model was constructed to describe the cancer‐adipose conversion. The ODEs have common forms as in Equation (1):

(1) $$\;\frac{dX}{dt}=g_{X}\cdot G-k_{X}\cdot K\cdot X$$

Here, *X* represents the level of gene expressions, *g_X_* and *k_X_* represent the basal synthesis and degeneration rates of *X(t)*, respectively, while *G* and *K* denote the regulation of other genes on the synthesis and the degradation of *X(t)*. Regulations among different components can be described by the product of the shifted Hill function: $Hs\;\left(Y,\;S,\;\lambda ,\;n\right)=\;1+\left(\lambda -1\right)\frac{Y^{n}}{S^{n}+Y^{n}\;}$.^[^ ^14^, ^76^ ^]^ Here, λ represents the fold change for the regulations, S represents the threshold of the sigmoidal function, and n is the Hill coefficient, which determines the steepness of the sigmoidal function. Y represents the regulator. The Hill function depends on λ in the following way:

(2) $$\mathrm{Hs}\left(Y,\;S,\;\lambda ,\;n\right)\left\{\begin{array}{c} <1,\lambda <1\\ =1,\;\lambda =1\\ >1,\;\lambda >1 \end{array}\right.$$

Here, the fold change λ decides whether this regulation was activation or inhibition (λ > 1 for activation and λ < 1 for inhibition) and the strength of regulations. The CAC network comprises a few subnetworks. The first subnetwork was the EMT process, which involves the reciprocal interaction between p53‐induced microRNAs and EMT transcription factors (ZEB1, SNAIL1). The second subnetwork was cancer metastasis process, which involves RKIP, Lin28, Let7, Bach1, EMT transcription factors and p53‐induced microRNAs. The third subnetwork was MAPK pathway and adipogenesis. MEK was inhibited by ERK, RKIP and other MEK inhibitor.^[^ ^75^ ^]^ ERK was activated by MEK and has both self‐activation and self‐inhibition effect.^[^ ^77^ ^]^ PPAR*γ* and C/EBP*α* form a positive feedback loop with each other.^[^ ^78^ ^]^ The three drugs corresponding to TGF‐*β*, MEKi and Rosiglitazone were also modeled by introducing three input nodes in the CAC network (Figure 1A, see Supporting Information for detailed model).

### Self‐Consistent Mean Field Approximation

The probability distribution $P(X_{1},X_{2},\text{…},X_{n},t)$ of a dynamical system was governed by probabilistic diffusion equations, where $X_{1},X_{2},\text{…},X_{n}$ represent concentration of proteins or gene expression levels in cells. To obtain the probability distribution of a gene regulatory network model, a self‐consistent mean field approach^[^ ^23^, ^34^, ^79^, ^80^ ^]^ was followed to split the probability into products of the individual ones, i.e., $P\left(X_{1},X_{2},\text{…},X_{n},t\right)∼\prod_{i}^{n}P\left(X_{i},t\right)$ and the probability was solved self‐consistently.

Diffusion equations were hard to solve directly for high‐dimensional systems, so it started from the moment equations instead. By assuming Gaussian distribution as an approximation, two moments were need to calculated, the mean and the variance. When the diffusion coefficient *D* was small, the moment equations can be approximated by:^[^ ^81^, ^82^ ^]^

(3) $$\dot{\bar{x}}\left(t\right)=F\left[\bar{x}\left(t\right)\right]$$

(4) $$\dot{\sigma }\;\left(t\right)=\sigma \left(t\right)A^{T}\left(t\right)+A\left(t\right)\sigma \left(t\right)+2D\left[\bar{x}\left(t\right)\right]$$

Here, **x**, **σ**(*t*), and **A**(*t*) are vectors and tensors, and **A^T^** (*t*) is the transpose of A(t). The elements of matrix **A** are specified as:

(5) $$\;A_{ij}=\frac{\partial F_{i}\left[X\left(t\right)\right]}{\partial x_{j}\left(t\right)}\;$$

Based on these equations, it can solve $\bar{x}\left(t\right)$ and **σ**(t). Here only, the diagonal elements of **σ**(*t*) were considered from the mean field approximation. Therefore, the evolution of probability distribution for each variable can be obtained from the Gaussian approximation:

(6) $$\begin{array}{c} P\;\left(x,t\right)=\frac{1}{\sqrt{2\pi \sigma \left(t\right)}}\;\exp-\frac{{\left[x-\bar{x}\left(t\right)\right]}^{2}}{2\sigma \left(t\right)} \end{array}$$

Here, $\bar{x}\left(t\right)$ and **σ**(*t*) are the solutions of Equations (3) and (4). The probability distribution obtained above corresponds to one stable state. If the system has multiple stable states, there should be several probability distributions localized at each basin with different variances. Thus, the total probability was the weighted sum of all these individual probability distributions. From the mean field approximation, this formulation can be extended to the multidimensional case by assuming that the total probability was the product of each individual probability for each variable. Finally, with the total probability, the potential landscape can be constructed by: *U* (*x*)= − *lnP_ss_* (*x*), with *P_ss_* (*x*) representing steady state probability distribution.^[^ ^22^, ^23^ ^]^

### Transition Paths and Landscape Control

A dynamical system in the fluctuating environments can be addressed by:

(7) $$\dot{\bar{x}}\left(t\right)=F\left[\bar{x}\left(t\right)\right]+\zeta$$

Here, $x\;=(x_{1}\left(t\right),x_{2}\left(t\right),\text{…}\;,\;x_{n}\left(t\right))$ represents the vector of the expression level of proteins or genes. **F**[**x**(*t*)] is the vector for the driving force from the dynamical system, ζ is the Gaussian white noise term, which satisfies E [ζ_*i*_(*t*)ζ_*j*_(0)] = 2*D*δ_*ij*_δ(*t*). Here, *D* is the constant diffusion coefficient characterizing the level of noise, δ(*t*) is Dirac Delta function, which means that the noises at different times were independent, and δ_*ij*_ satisfies:

(8) $$\begin{array}{c} \left\{\begin{array}{c} \delta_{\mathrm{ij}}=1,i=j\\ \delta_{\mathrm{ij}}=0,i\neq j \end{array}\right. \end{array}$$

Following the approaches^[^ ^33^, ^55^ ^]^ based on the Freidlin–Wentzell theory,^[^ ^83^ ^]^ the most probable transition path from attractor *i* at time 0 to attractor *j* at time T, can be acquired by minimizing the action functional over all possible paths:

(9) $$\begin{array}{c} S_{T}\;\left[\varphi_{ij}\right]=\frac{1}{2}\;\int_{0}^{T}|{\dot{\varphi }}_{ij}-F\left(\varphi_{ij}\right){|}^{2}dt \end{array}$$

This path was called the minimized action path (MAP). MAPs were calculated numerically by applying minimum action methods.^[^ ^55^ ^]^

To identify the optimal combination of drugs for promoting CAC, the landscape control method for the CAC model^[^ ^8^, ^33^, ^63^ ^]^ was employed. Here, our goal was to predict therapeutic targets (189 parameters characterizing synthesis rate, degradation rate and interaction intensity etc., see Supporting Information for details) that can promote the transition from E, M, and partial EMT state to the A state. As such, the optimization process was to minimize the transition action from E, M, and partial EMT state to the A state and maximize the transition action from A to E, M, and partial EMT state (smaller transition action means larger transition probability), by tuning each of 189 parameters. To this end, a cost function was defined for maximizing the occupancy of the A state as ΔS_A_ = (S_M→A_‐S_A→M_) + (S_E→A_‐S_A→E_) + (S_P1→A_‐S_A→P1_) + (S_P2→A_‐S_A→P2_), and our aim was to minimize ΔS_A_. The Adaptive Minimum Action Method^[^ ^55^ ^]^ was used to calculate the transition action, and the matlab function “fmincon” to perform the minimization of transition actions.

### Cell Culture

The breast cancer cell line (MDA‐MB‐231), hepatoma cell line (Huh‐7 and Hep3B), and colon cancer cell line (SW480 and SW620) were obtained from the Cell Bank of the Chinese Academy of Sciences (Shanghai, China), and cultured at 37 °C in 5% CO_2_.

### Western Blot

Cells were lysed in RIPA buffer containing 1 mm PMSF (Solarbio, Beijing, China). The protein concentration was measured using BCA Protein Assay Kit (Thermo Fisher Scientific). Protein samples were boiled and then separated on 8–10% SDS‐PAGE gels, followed by transfer on polyvinylidene fluoride (PVDF) membranes (Millipore). These membranes were blocked for 1 h in 5% (w/v) skimmed milk at room temperature and then incubated at 4 °C with primary antibody overnight. After washing with TBS‐T three times, the membranes were incubated with horseradish peroxidase (HRP)‐conjugated secondary antibody for 1 hour at room temperature. Finally, the blots were visualized with enhanced chemiluminescence (ECL) reagent (Millipore).

### qRT‐PCR

Total RNA was isolated from cells by using mirVana miRNA Isolation Kit (Life Technologies, Grand Island, NY, USA) or TRIzol Reagent (Life Technologies) according to the standard protocol. For miRNA, reverse transcriptions were performed using the TaqMan miRNA Reverse Transcription Kit (Life Technologies), and cDNA amplification was performed using the TaqMan miRNA Assay Kit (Life Technologies) according to the manufacturer's instructions. The expression of mRNA was determined using the GoTaq qPCR Master Mix (Promega, Madison, WI, USA), with actin used as the endogenous control. Gene expression fold changes were assessed using the 2DCt method. The primers used are listed as follows.

**Table:** 

Pparg2	GCTGTGAAGTTCAATGCACTGG	GCAGTAGCTGCACGTGCTCTG
Klf4	CGGGAAGGGAGAAGACACT	GAGTTCCTCACGCCAACG
C/EBPa	AAACAACGCAACGTGGAGA	GCGGTCATTGTCACTGGTC
Zeb1	GCCAGCAGTCATGATGAAAA	TATCACAATACGGGCAGGTG
Snail1	CTCTGAAGATGCACATCCGAA	GGCTTCTCACCAGTGTGGGT
*β*‐actin	TCCCTGGAGAAGAGGCTACGA	AGGAAGGAAGGCTGGAAGAG

### Wound‐Healing Assay

Cells were cultured and grown to 90% confluence in six‐well plates and then cultured overnight in serum‐free medium. The cell wound was drawn by a 10 mL pipette tip in a straight line. After washing with PBS, wound healing images were taken immediately via an inverted microscope imaging system (Olympus). Then cells were then cultured in medium containing 1% FBS for 24 h. The 24 h images were taken in the same way.

### Colony‐Formation Assays

For a colony‐formation assay, 500 cells were seeded in six‐well plates. After incubation at 37 °C ≈3 weeks, the colonies were fixed with 4% paraformaldehyde (PFA) for 30 min at room temperature and stained with 0.2% crystal violet for 15 min. The number of visible colonies were counted using Adobe Photoshop (version 2020).

### Oil Red O Staining

The cells in each group were washed twice with PBS, fixed with 4% paraformaldehyde for 10 min, stained with oil red O solution for 10 min, and then restained with hematoxylin solution for 5 min. After washing, under a light microscope, the red granular material in the cytoplasm was the lipid in the cytoplasm stained with oil red O solution, and the nucleus was blue.

### Bodipy Dye

The cells to be stained were removed from the intercellular area and cleaned with PBS before fixation. After fixation, Bodipy working solution was added to stain for 30–60 min, and the cell culture plate was wrapped with tinfoil paper to avoid light. After staining, the cells were washed with PBS for three times, DAPI working solution was added to stain the nuclei, and PBS was cleaned again for three times, and fluorescence microscope was used to take photos.

### Flow Cytometric Analysis

Hep3B cells were seeded at a density of 1.5 × 105 cells per well in six‐well plates, grown for 20 h, and then pre‐incubated for 24 h under ZEB1 or MDM2 with ROSi conditions prior to incubation with BODIPY. After incubation for 30 min, the cells were gently scraped, suspended in PBS (Gibco), and transferred to flow cytometry tubes. Subsequently, the cells were analyzed using a flow cytometer (Attune NxT, Thermo Fisher) for Alexa Fluor 488. All analyses were carried out in triplicate using at least 10000 cells.

### Statistical Analysis

Data were shown as the mean with standard error. The one‐way ANOVA or Student's t test was used to determine differences between groups. A p‐value < 0.05 was considered statistically significant, and calculations were performed with Statistical Package for Social Science (SPSS for Windows, version 22; Chicago, IL, USA).

## Conflict of Interest

The authors declare no conflict of interest.

## Author Contributions

Z.C. and J.L. contributed equally to this work. C.L. designed research. Z.C. and J.L. performed research. Z.C., J.L., X.Z., H.Y., and C.L. analyzed data. Z.C., J.L., H.Y., and C.L. wrote the paper.

## Supporting information



Supporting Information

## Data Availability

The data that support the findings of this study are available in the supplementary material of this article.
