# Supplementary material for: Energy Landscape Reveals the Underlying Mechanism of Cancer‐Adipose Conversion in Gene Network Models
Source: Adv Sci (Weinh). 2024 Sep 11;11(41):2404854. doi: 10.1002/advs.202404854 (PMC11538663; doi:10.1002/advs.202404854)
Supplement: Supplementary file 1 — Supporting Information [file ADVS-11-2404854-s001.pdf]

ADVANCED SCIENCE
Open Access

Supporting Information

for *Adv. Sci.*, DOI 10.1002/advs.202404854

# Energy Landscape Reveals the Underlying Mechanism of Cancer-Adipose Conversion in Gene Network Models

*Zihao Chen, Jia Lu, Xing-Ming Zhao, Haiyang Yu* and Chunhe Li**

# Supplementary Information for

## Energy landscape reveals the underlying mechanism of cancer-adipose conversion in gene regulatory network models

**Zihao Chen, Jia Lu, Xing-Ming Zhao, Haiyang Yu, Chunhe Li**

# This PDF file includes:

**Supplementary text**

**Figures S1 to S17**

**Tables S1 to S6**

**SI References**

# Mathematical models

We constructed a cancer-adipose conversion (CAC) model based on the underlying gene regulatory network (Figure 1A in main text), from literature mining (see Table S1 for experimental evidences of regulations in the network). The CAC network contains multiple subcircuits, including the EMT process, cancer metastasis process, MAPK pathway, and adipogenesis process. To understand the dynamics of cancer-adipose conversion, we formulated a mathematical model using ordinary differential equations (ODEs). Our mathematical model consists of 16 components and 52 regulations (Table S1). The general form of each equation in the deterministic ODEs model is:

$$\frac{dX}{dt} = g_X \cdot G - k_X \cdot K \cdot X \tag{1}$$

Here, $X$ represents the expression level of each gene. $g_X$ and $k_X$ denote the basal synthesis rate and degradation rate of $X(t)$, individually. $G$ and $K$ denote the regulation of other genes on the synthesis and the degradation of $X(t)$. The regulations among different components can be described by the product of the shifted Hill function: $Hs(Y, S, \lambda, n) = 1 + (\lambda - 1)\frac{Y^n}{S^n+Y^n}$ [5,8]. Here, $\lambda$ represents the fold change for the regulations, $S$ represents the threshold of the sigmoidal function, and $n$ is the Hill coefficient, which determines the steepness of the sigmoidal function. $Y$ represents the regulator. The Hill function depends on $\lambda$ in the following way:

$$Hs(Y, S, \lambda, n) \begin{cases} < 1 & \lambda < 1 \\ = 1 & \lambda = 1 \\ > 1 & \lambda > 1 \end{cases} \tag{2}$$

which indicates that the fold change $\lambda$ decides whether this regulation is activation or inhibition ($\lambda > 1$ for activation and $\lambda < 1$ for inhibition) and the strength of regulations. Taking the activation effect of SNAI1 on ZEB1 as an example, the equation describing the evolution of the ZEB1 level takes the form as:

$$\begin{aligned}\frac{d(ZEB1)}{dt} =& g_z * Hs(SNAIL1, S_{SZ}, \lambda_{SZ}, n_{SZ}) * Hs(ZEB1, S_{ZZ}, \lambda_{ZZ}, n_{ZZ}) * TGF- \\ & k_z * Hs(miR200, S_{m2Z}, \lambda_{m2Z}, n_{m2Z}) * Hs(SNAIL1, S_{m1Z}, \lambda_{m1Z}, n_{m1Z}) * ZEB1\end{aligned} \tag{3}$$

The ODE models for different subcircuits are introduced below, and the meaning of each parameter is

described in Table S2 and S3.

**EMT transcription factors and P53-induced microRNAs**

In our model, the EMT process can be understood as the reciprocal switch between P53-induced microRNAs and EMT transcription factors. EMT transcription factors including SNAI1 and ZEB family inhibit E-cadherin (Cdh1) and induce EMT process [17, 18]. ZEB1/2 can be activated by SNAI1 and itself, while SNAI1 self-inhibits itself.

$$\begin{aligned}\frac{d(SNAIL1)}{dt} =& g_{_S} * Hs(SNAIL1, S_{SS}, \lambda_{SS}, n_{SS}) * TGF - k_{_S} * Hs(miR34, S_{m3S}, \lambda_{m3S}, n_{m3S}) \\ & * Hs(Let, S_{LetS}, \lambda_{LetS}, n_{LetS}) * Hs(RKIP, S_{RS}, \lambda_{RS}, n_{RS}) * SNAIL1,\end{aligned} \tag{4}$$

P53, an important tumor suppressor gene, activates microRNAs (miR145, miR200 and miR34) to inhibit the EMT transcription factors (SNAI1 and ZEB1/2), while the EMT transcription factors hamper the effects of P53-induced microRNAs [10]. The oncogene MDM2, which is induced by P53, is able to inhibit P53 functions [24]. P53 inhibits OCT4 in both direct way and indirect way by promoting miR145 [9,14]. OCT4 inhibits miR145 while activates miR200 [25].

$$\frac{d(p53)}{dt} = g_p * G_{p53} - k_p * Hs(MDM2, S_{MDp}, \lambda_{MDp}, n_{MDp}) * p53 \tag{5}$$

$$\begin{aligned}\frac{d(OCT4)}{dt} =& g_{_O} * Hs(p53, S_{pO}, \lambda_{pO}, n_{pO}) * Hs(Lin, S_{LinO}, \lambda_{LinO}, n_{LinO}) - k_{_O} * Hs(OCT4, S_{OO}, \lambda_{OO}, n_{OO}) \\ & * Hs(miR145, S_{m1O}, \lambda_{m1O}, n_{m1O}) * OCT4\end{aligned} \tag{6}$$

$$\frac{d(MDM2)}{dt} = g_{_{MD}} * Hs(p53, S_{pMD}, \lambda_{pMD}, n_{pMD}) - k_{_{MD}} * Hs(miR145, S_{m1MD}, \lambda_{m1MD}, n_{m1MD}) * MDM2 \tag{7}$$

$$\begin{aligned}\frac{d(miR145)}{dt} =& g_{m1} * Hs(p53, S_{pm1}, \lambda_{pm1}, n_{pm1}) - k_{m1} * Hs(ZEB1, S_{Zm1}, \lambda_{Zm1}, n_{Zm1}) \\ & * Hs(OCT4, S_{Om1}, \lambda_{Om1}, n_{Om1}) * miR145\end{aligned} \tag{8}$$

$$\frac{d(miR200)}{dt} = g_{m2} * Hs(p53, S_{pm2}, \lambda_{pm2}, n_{pm2}) * Hs(OCT4, S_{Om2}, \lambda_{Om2}, n_{Om2}) - k_{m2} * Hs(ZEB1, S_{Zm2}, \lambda_{Zm2}, n_{Zm2}) * Hs(SNAIL1, S_{Sm2}, \lambda_{Sm2}, n_{Sm2}) * miR200 \tag{9}$$

$$\frac{d(miR34)}{dt} = g_{m3} * Hs(p53, S_{pm3}, \lambda_{pm3}, n_{pm3}) - k_{m3} * Hs(ZEB1, S_{Zm3}, \lambda_{Zm3}, n_{Zm3}) * Hs(SNAIL1, S_{Sm3}, \lambda_{Sm3}, n_{Sm3}) * miR34 \tag{10}$$

**Cancer metastasis process**

For the metastasis circuit, OCT4 induces Let7, while Let7 inhibits the LIN28 [25]. Let7 and LIN28 form a dual-negative feedback loop [28]. Let7 inhibits both SNAI1 and BACH1. BACH1 promotes the metastasis through facilitating MMP1 [3]. In breast cancer cells, BACH1 inhibits the transcription of the Raf kinase inhibitor protein (RKIP), which forms a feedback loop between Let7, BACH1 and RKIP involving that RKIP activates Let7 [2,12].

$$\frac{d(RKIP)}{dt} = g_{RK} * Hs(BACH1, S_{BR}, \lambda_{BR}, n_{BR}) - k_{RK} * Hs(SNAIL1, S_{SR}, \lambda_{SR}, n_{SR}) * RKIP \tag{11}$$

$$\frac{d(Let7)}{dt} = g_{Let} * Hs(miR200, S_{m2Let}, \lambda_{m2Let}, n_{m2Let}) * Hs(RKIP, S_{RLet}, \lambda_{RLet}, n_{RLet}) - k_{Let7} * Hs(LIN28, S_{LinLet}, \lambda_{LinLet}, n_{LinLet}) * Hs(Let7, S_{LetLet}, \lambda_{LetLet}, n_{LetLet}) * Let7 \tag{12}$$

$$\frac{d(LIN28)}{dt} = g_{Lin} * Hs(MEK, S_{MLin}, \lambda_{MLin}, n_{MLin}) * Hs(OCT4, S_{OLin}, \lambda_{OLin}, n_{OLin}) * (RKIP, S_{RLin}, \lambda_{RLin}, n_{RLin}) - k_{Lin} * Hs(LIN28, S_{LinLin}, \lambda_{LinLin}, n_{LinLin}) * Hs(Let7, S_{LetLin}, \lambda_{LetLin}, n_{LetLin}) * Hs(miR200, S_{m2Lin}, \lambda_{m2Lin}, n_{m2Lin}) * LIN28 \tag{13}$$

$$\frac{d(BACH1)}{dt} = g_{B} * Hs(BACH1, S_{BB}, \lambda_{BB}, n_{BB}) - k_{B} * Hs(Let7, S_{LetB}, \lambda_{LetB}, n_{LetB}) * BACH1 \tag{14}$$

**MAPK pathway and adipogenesis**

BMP2, a member of the TGF$\beta$ super family, has been noted to enhance the adipogenesis potential of mesenchymal stem cells [4,16]. However, TGF$\beta$ also promotes the metastasis in malignant tumor through activation of MEK and EMT transcription factors [17,18]. Rosiglitazone significantly amplifies the expression of adipogenesis markers, including peroxisome proliferator-activated receptor gamma (PPAR$\gamma$) and CCAAT/enhancer-binding protein $\alpha$ (C/EBP$\alpha$) [21]. MEK activates the PPAR$\gamma$ and C/EBP$\alpha$ [19], while ERK inhibits C/EBP$\alpha$ and PPAR$\gamma$ [20]. Besides, MEK forms a positive feedback loop through activation of Raf [27]. PPAR$\gamma$ and C/EBP$\alpha$ are able to form a positive feedback loop through activating each other [29].

$$\begin{aligned}\frac{d(MEK)}{dt} =& g_{MEK} * Hs(MEK, S_{MM}, \lambda_{MM}, n_{MM}) * TGF - k_{MEK} * Hs(RKIP, S_{RM}, \lambda_{RM}, n_{RM}) \\ & * Hs(ERK, S_{EEi}, \lambda_{EM}, n_{EM}) * MEK * MEKi\end{aligned} \tag{15}$$

$$\begin{aligned}\frac{d(ERK)}{dt} =& g_{ERK} * Hs(MEK, S_{ME}, \lambda_{ME}, n_{ME}) * Hs(ERK, S_{EE}, \lambda_{EE}, n_{EE}) \\ & - k_{ERK} * Hs(ERK, S_{EEi}, \lambda_{EEi}, n_{EEi}) * ERK * Rosi\end{aligned} \tag{16}$$

$$\begin{aligned}\frac{d(CEBP)}{dt} =& g_{CEBP} * Hs(MEK, S_{MC}, \lambda_{MC}, n_{MC}) * Hs(PPAR, S_{PC}, \lambda_{PC}, n_{PC}) * Rosi \\ & - k_{CEBP} * Hs(ERK, S_{EC}, \lambda_{EC}, n_{EC}) * TGF * CEBP\end{aligned} \tag{17}$$

$$\begin{aligned}\frac{d(PPAR)}{dt} =& g_{PPAR} * Hs(MEK, S_{MP}, \lambda_{MP}, n_{MP}) * Hs(CEBP, S_{CP}, \lambda_{CP}, n_{CP}) * Rosi \\ & - k_{PPAR} * Hs(ERK, S_{EP}, \lambda_{EP}, n_{EP}) * PPAR\end{aligned} \tag{18}$$

## Parameter setting for the models

1. Our model contains 189 parameters, including synthesis rate, degradation rate, interaction intensity, threshold, and Hill coefficient for each regulation. To reduce the number of the tunable parameters, we set the parameters uniformly based on their interaction types. For example, we set all the synthesis rate ($g_x$) to $a$, degradation rate ($k_x$) to $d$, threshold to $L$, and Hill coefficient to $n$, respectively. We set the interaction intensity ($\lambda$) to $\lambda_a$ ($\lambda > 1$) or $\lambda_i$ ($0 < \lambda < 1$) according to the interaction type ($\lambda > 1$ for activation and $0 < \lambda < 1$ for inhibition), respectively. In this way, we reduce the parameter number to 6 (Table S2). We also have three tunable parameters describing three drugs including TGF-$\beta$, MEKi (MEK inhibitor), and Rosi (Rosiglitazone).

2. Previous studies showed that the EMT process involves at least three stable states (epithelial, mesenchymal, and partial EMT states) [13, 15]. Therefore, a reasonable model involving EMT process should be able to generate more than three stable states, and these stable states should match the phenotypes in EMT process in gene expression level of EMT markers (e.g., EMT transcription factors, P53-induced microRNAs). Therefore, we need to search for a set of parameters in a high-dimensional gene network system which can generate multiple (larger than three) stable states. Here we used a random parameter searching approach given a range for each parameter:

First, following previous work [5, 8, 15], we set the searching range for different parameters as: a (0, 20), d (0, 2), $\lambda_a$ (1, 20), $\lambda_i$ (0, 1), n (2, 6), L (0, 1000), TGF-$\beta$ (1, 10), MEKi (0, 1.2), Rosi (0, 30) and the initial partition length for a (1), d (0.1), $\lambda_a$ (1), $\lambda_i$ (0.1), n (1), L (100), TGF-$\beta$ (0.1), MEKi (0.01), and Rosi (0.5).

Then we start from a random combination of the parameters and perform parameter searching by checking the number of stable states with this set of parameters. For each of the parameter, we set the total searching steps as 5. Within the searching steps, if the number of the stable states increase, we update the parameter values and reset the searching steps to 0. We do this one parameter by one.

If all the parameters are visited, then reset the initial parameter range as the collection of adjacent partitions and divide a finer mesh in the adjacent partition of the optimal parameters. To reduce the computation cost, we use a hash table to count the partitions which have been visited already. To avoid the local optimal results, we try different combinations of initial parameters and record the parameters

corresponding to the system with more than three attractors.

3. After above parameter searching process, we obtain some typical parameter values for six unified parameters and three drugs (TGF-$\beta$, MEKi, Rosi) that can generate multiple attractors (more than three attractors, see Table S2). Then we use the expression level of EMT markers to distinguish the phenotype of each attractor and obtain the parameter set with attractors corresponding to E, M, and partial EMT states in EMT process (first row in Table S2).

4. We further tune the parameters more specifically to make our model resemble the experimental procedure for drug addition process in previous study [7]. The detailed tuned parameter values are shown in Table S3. In this way, we constructed a more robust model which can generate five stable states (E, M, P1, P2, A), and which can replicate the experimental observation for drug additions.

5. We realize that the intensity of interaction ($\lambda$ and the threshold of each regulation) in CAC network may be different for different regulations in realistic systems. Therefore, we performed a sensitivity analysis by varying the parameters corresponding to different regulations (links) to identify the effects of different regulations on the transition dynamics (Figure S11-S13), as well as testing the robustness of the model against these parameters. We showed both local (Table S3) and global (Figure S11-S13) sensitivity analysis for our model, which support the robustness of our model against the parameter perturbations.

## Integration of negative feedback loop of TGF$\beta$ to the model

Our modeling framework allows for integrating multiple feedback loops between genes or pathways, as long as we have the network structure. In our CAC network, we have not considered the negative feedback loops of TGF-$\beta$ on itself. But, our model can be easily extended to include this feedback loop. For example, in above model, we use TGF$\beta_{total}$ to denote the level of TGF-$\beta$. Now we integrated the negative feedback loops of TGF-$\beta$ into CAC process by changing the previous parameters of TGF-$\beta_{total}$ to the variables TGF-$\beta$ with the formulation:

$$\frac{d(TGF\beta)}{dt} = TGF\beta_{total} - TGF\beta * (1 - \frac{1}{exp(\frac{TGF\beta}{I})}) \tag{19}$$

Here we keep other parameters in the model unchanged, and let $TGF\beta_{total} \cdot I = 1$, where $I$ is the normalizing factor for unifying the units. In this updated model with negative feedback of TGF-$\beta$, we found four stable states on the landscape, including E, P1, P2 and A state (Figure S16). M state disappears with the gradual decrease in TGF-$\beta$ concentration. We also see that the TGF-$\beta$ level decreases with time caused by the negative feedback loops. These results demonstrate that our model can be modified to consider multiple feedback structures.

# Supplemental methods

## Transcript analyses

For RNA-Seq analysis, total amount and integrity of RNA were evaluated utilizing the RNA Nano 6000 Assay Kit of the Bioanalyzer 2100 system (Agilent Technologies, CA, USA). Total RNA served as the input material for the RNA sample preparations. The mRNA was purified from the total RNA using poly-T oligo-attached magnetic beads. Fragmentation of purified mRNA was achieved by divalent cations under elevated temperature in First Strand Synthesis Reaction Buffer(5X). Subsequently, first strand cDNA was synthesized utilizing random hexamer primer and M-MuLV Reverse Transcriptase. RNA degradation was carried out using RNaseH. Next, the second strand cDNA was synthesized using DNA Polymerase I and dNTP. The remaining overhangs were converted into blunt ends through exonuclease/polymerase activities. The DNA fragments' 3' ends were adenylated, followed by ligation of an Adaptor with a hairpin loop structure to prepare for hybridization. Purification of the library fragments with preferentially 150-200 bp in length was achieved utilizing the AMPure XP system (Beckman Coulter, Beverly, USA). After PCR amplification, the PCR product was purified by AMPure XP beads, and the library was obtained. The quality of the library was ensured via testing. Following the construction of the library, initial quantification of the library was carried out using Qubit2.0 Fluorometer, diluted to 1.5ng/$\mu$L, and the library's insert size was detected by Agilent 2100 bioanalyzer after the insert size met the expectations. To guarantee the library's quality, qRT-PCR was utilized to accurately quantify the library's effective concentration (higher than 2nM).

## Clustering and sequencing

After qualification of the library, different libraries were pooled according to their effective concentration and the target amount of data required. Subsequently, the pooled libraries were subjected to sequencing via the Illumina NovaSeq 6000. Sequencing was carried out using the basic principle of synthesizing and sequencing simultaneously, known as Sequencing by Synthesis. Four fluorescent labeled dNTP, DNA polymerase, and splice primers were added to the sequenced flowcell and amplified. Upon the extension of the complementary chain by the sequence cluster, each fluorescence-labeled dNTP can release its corresponding fluorescence. The sequencer captures the fluorescence signal and converts the optical signal into the sequencing peak through computer software, which generates the sequence information of the fragment to be tested. The end reading generated by the sequencing process is a 150bp pairing.

## Data analysis

The image data produced by the high-throughput sequencer were processed into sequence data (reads) using CASAVA base recognition. The raw data (raw reads) obtained in fastq format was processed through the fastp software to generate clean data (clean reads) by removing reads containing adapters, N base, and low-quality reads from the raw data. The quality of the clean data was evaluated based on Q20, Q30, and GC content. All downstream analyses were performed using high-quality clean data. The reference genome and gene model annotation files were downloaded from the genome website, and the index of the reference genome was generated using Hisat2 (v2.0.5). The paired-end clean reads were aligned to the reference genome using Hisat2 (v2.0.5). We selected Hisat2 as the mapping tool due to its ability to generate a database of splice junctions based on the gene model annotation file, thereby achieving a better mapping result compared to non-splice mapping tools.

## Reads mapping to the reference genome

The reference genome and gene model annotation files were acquired from genome website directly. Subsequently, an index of the reference genome was constructed using Hisat2 (v2.0.5), and paired-end clean reads were aligned to the reference genome using the same software. The selection of Hisat2 as the mapping tool was based on its capability to generate a database of splice junctions using the gene model annotation file. This feature resulted in better mapping results compared to non-splice mapping tools.

## Quantification of gene expression level

The software featureCounts (v1.5.0-p3) was employed to quantify the number of reads mapped to each gene. The Fragments Per Kilobase of transcript per Million mapped reads (FPKM) metric was computed for each gene using the gene length and read count information. FPKM accounts for sequencing depth and gene length simultaneously, and is used to estimate gene expression levels.

## Differential expression analysis

Differential expression analysis of two conditions/groups (two biological replicates per condition) was performed using the DESeq2 R package (1.20.0). DESeq2 provides statistical routines for determining differential expression in digital gene expression data using a model based on the negative binomial distribution. The resulting p-values were adjusted using the Benjamini and Hochberg's approach for controlling the false discovery rate. $padj \leq 0.05$ and $|log2(foldchange)| \geq 1$ were set as the threshold for significantly differential expression.

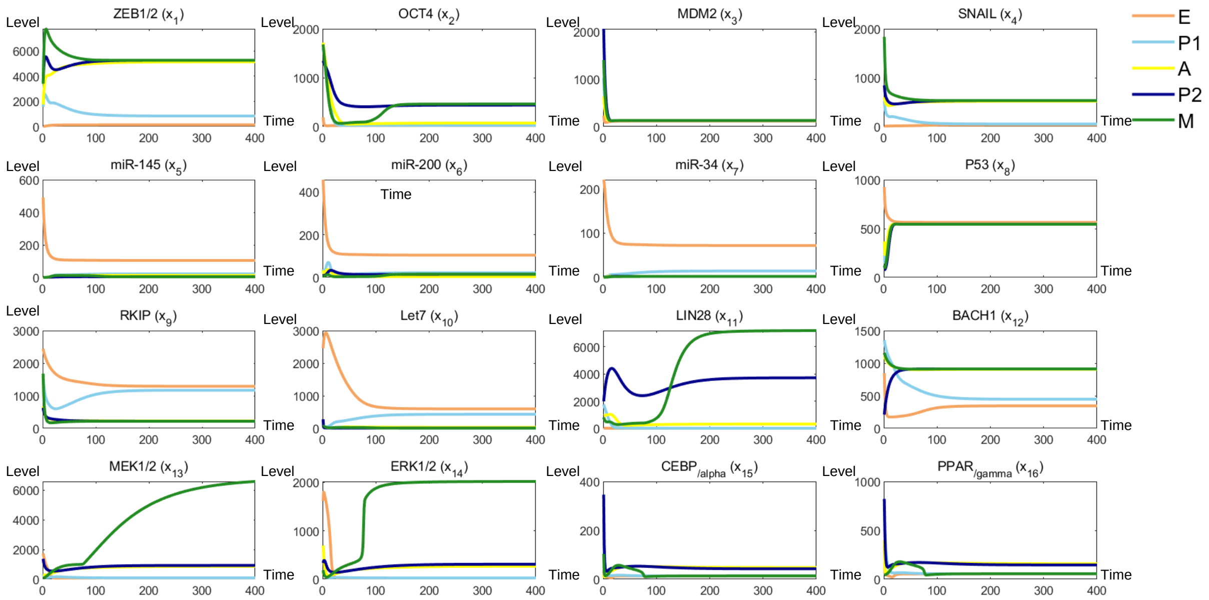


**Figure S1. The ODE simulation results with five stable states.** The simulation trajectories from the ODE model with five stable states are shown in terms of the 16 variables. Different trajectory represents different stable state. E represents epithelial state, M represents mesenchymal state, A represents the adipose state, and P1/P2 represent the partial EMT states.

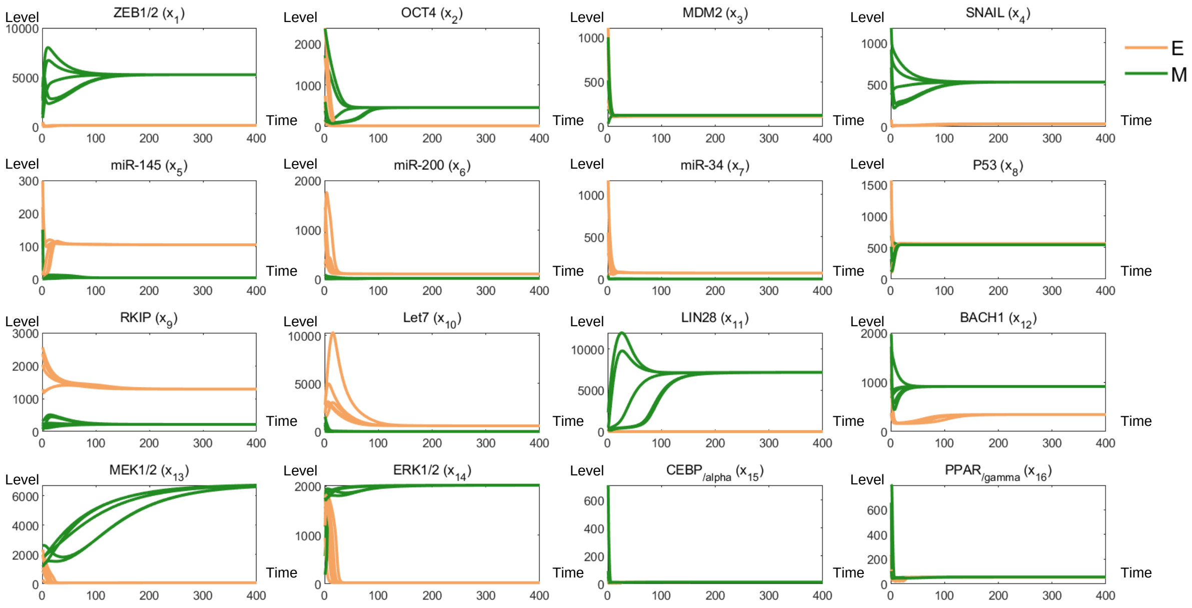


**Figure S2. The ODE simulation results for E and M state with different initial conditions.** Multi-state simulation trajectories of E and M states using 16 variables under five different initial conditions. Same color trajectories represent the same stable state. E and M denote epithelial and mesenchymal states, respectively.

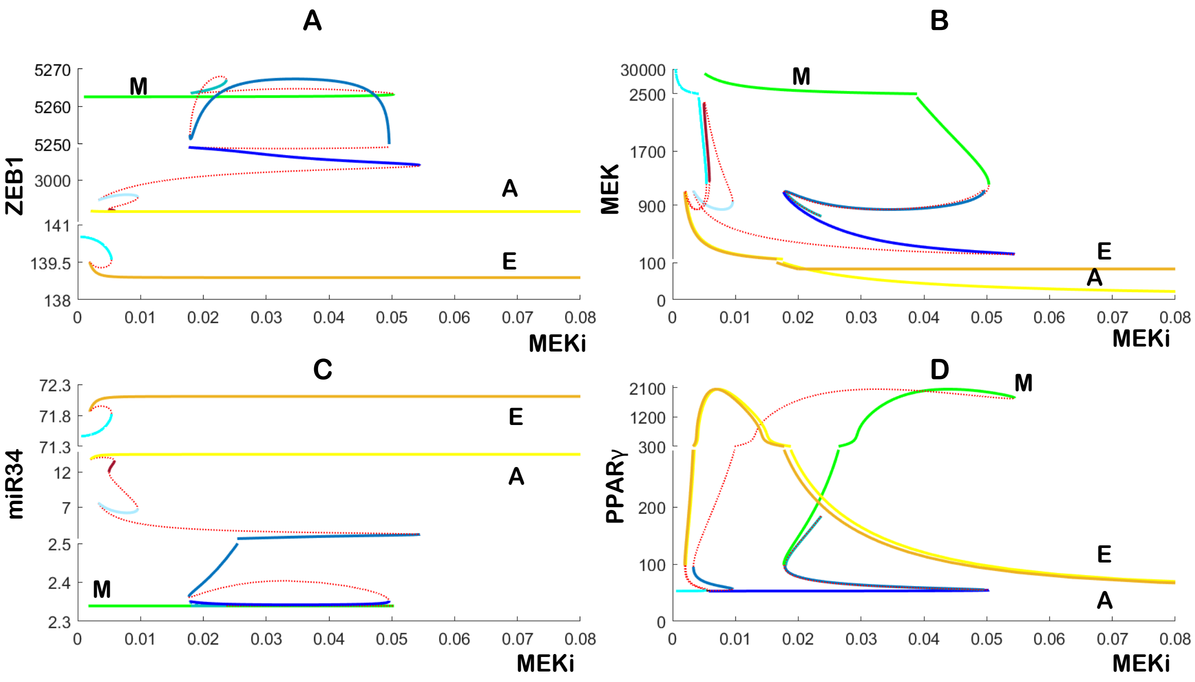


**Figure S3. Bifurcation analysis of MEKi with respect to the four variables.** (A) Bifurcation analysis of MEKi with respect to ZEB1. Solid lines represent the stable states, while red dotted lines represent the unstable states. Epithelial, mesenchymal, and adipose states are denoted by E, M, and A, respectively. (B) Bifurcation analysis of MKEi with respect to MEK. (C) Bifurcation analysis of MEKi with respect to miR34. (D) Bifurcation analysis of MEKi with respect to PPAR$\gamma$.

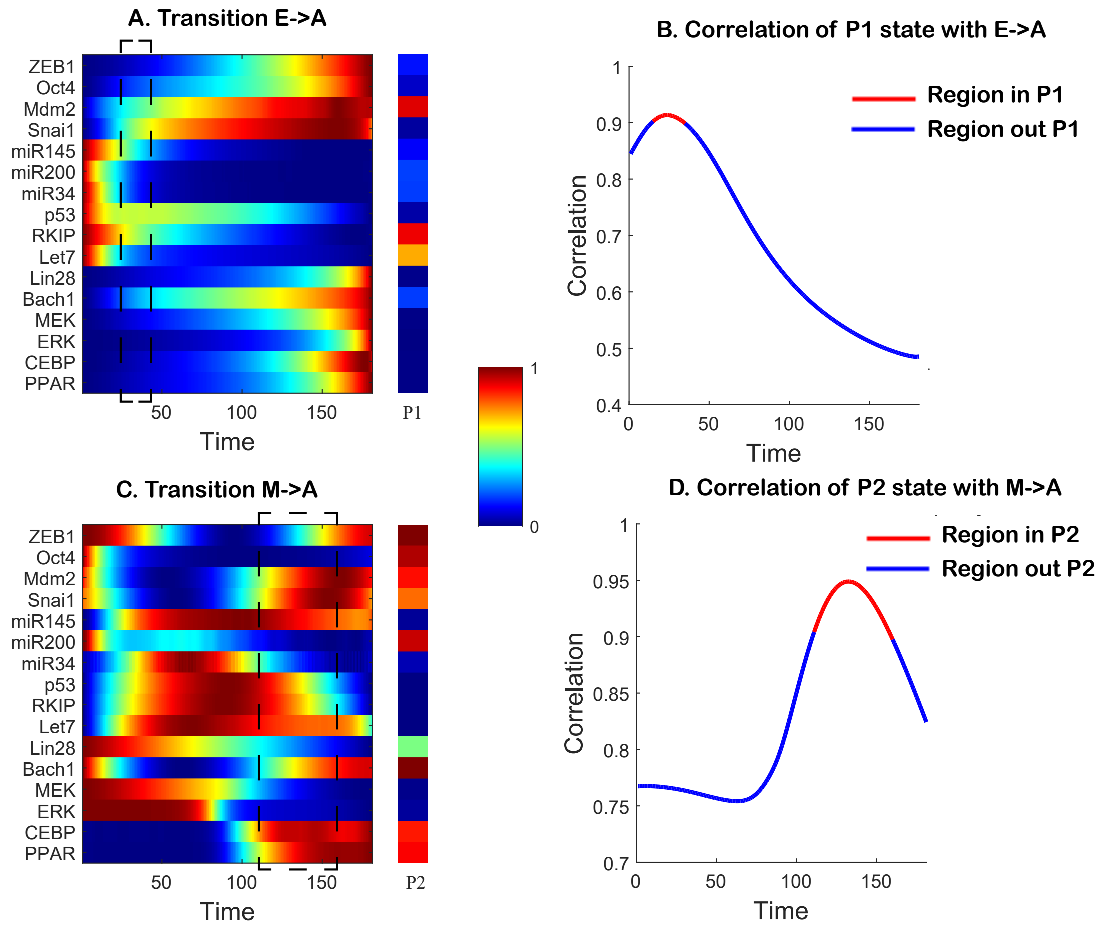


**Figure S4. Identification of partial EMT/intermediate states using transition path analysis from E to A and M to A state.** (A) Transition path analysis for the E to A transition. The color bars represent normalized expression level, "0" represents inactivated states, and "1" represents activated states. The "Time" axis represents the pseudo-time of each transition and the Y axis represents different gene expressions. The black dashed boxes indicate the regions close to the intermediate states P1. The column "P1" represents the expression level of P1. (B) Pearson correlation results between the expression level along transition path from E to A and the stable P1 state. Red line represents the identified region (corresponding to the region marked with the black dashed box in A) has higher correlation with P1 stable state. (C) Transition path for the transition from M to A. The region marked with the black dashed box is the region (denoted as P2 region) identified to be close to the P2 intermediate state. The column "P2" represents the expression level for P2 stable state from the model with 5 stable states. (D) Pearson correlation results between the expression level along transition path from M to A and the stable P2 state. Red line represents the identified region (corresponding to the region marked with the black dashed box in C) has higher correlation with P2 stable state.

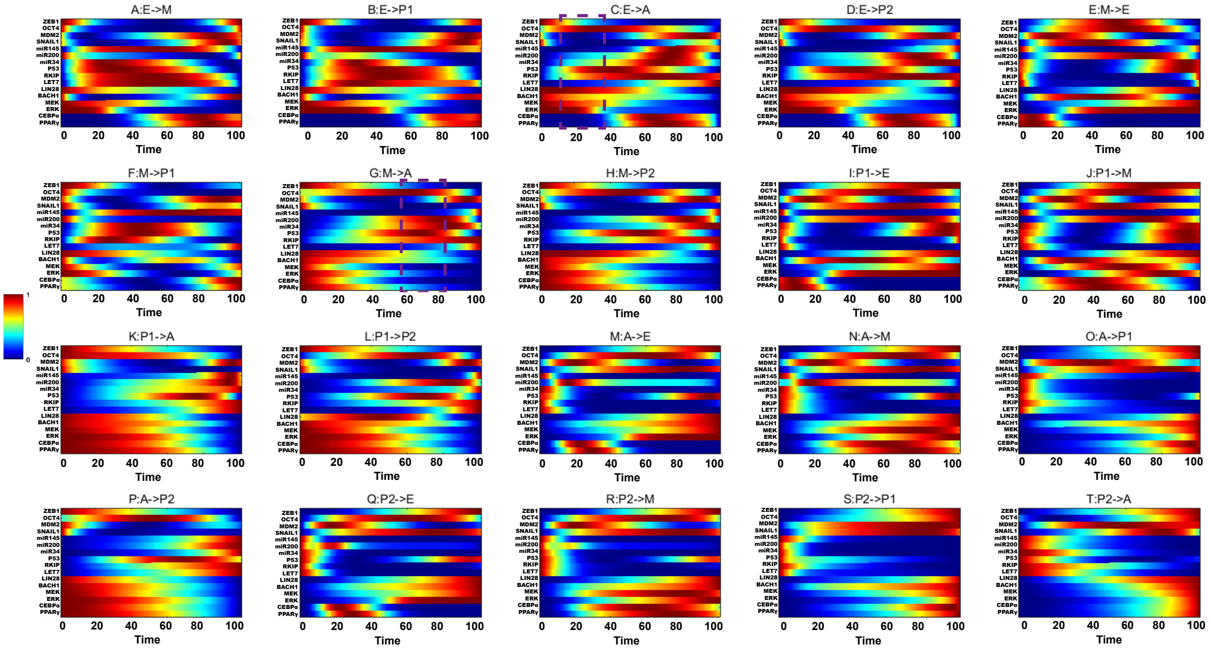


**Figure S5. Analysis of transition paths among five stable states.** (A) Transition path for the transition from E to M. The color bars represent normalized expression level with "0" representing inactivated states, and "1" representing activated states. The "Time" axis represents the pseudo-time of each cell state transition and the Y axis represents different gene expression levels. We show (B) E to P1, (C) E to A, (D) E to P2, (E) M to E, (F) M to P1, (G) M to A, (H) M to P2, (I) P1 to E, (J) P1 to M, (K) P1 to A, (L) P1 to P2, (M) A to E, (N) A to M, (O) A to P1, (P) A to P2, (Q) P2 to E, (R) P2 to M, (S) P2 to P1, and (T) P2 to A transition.

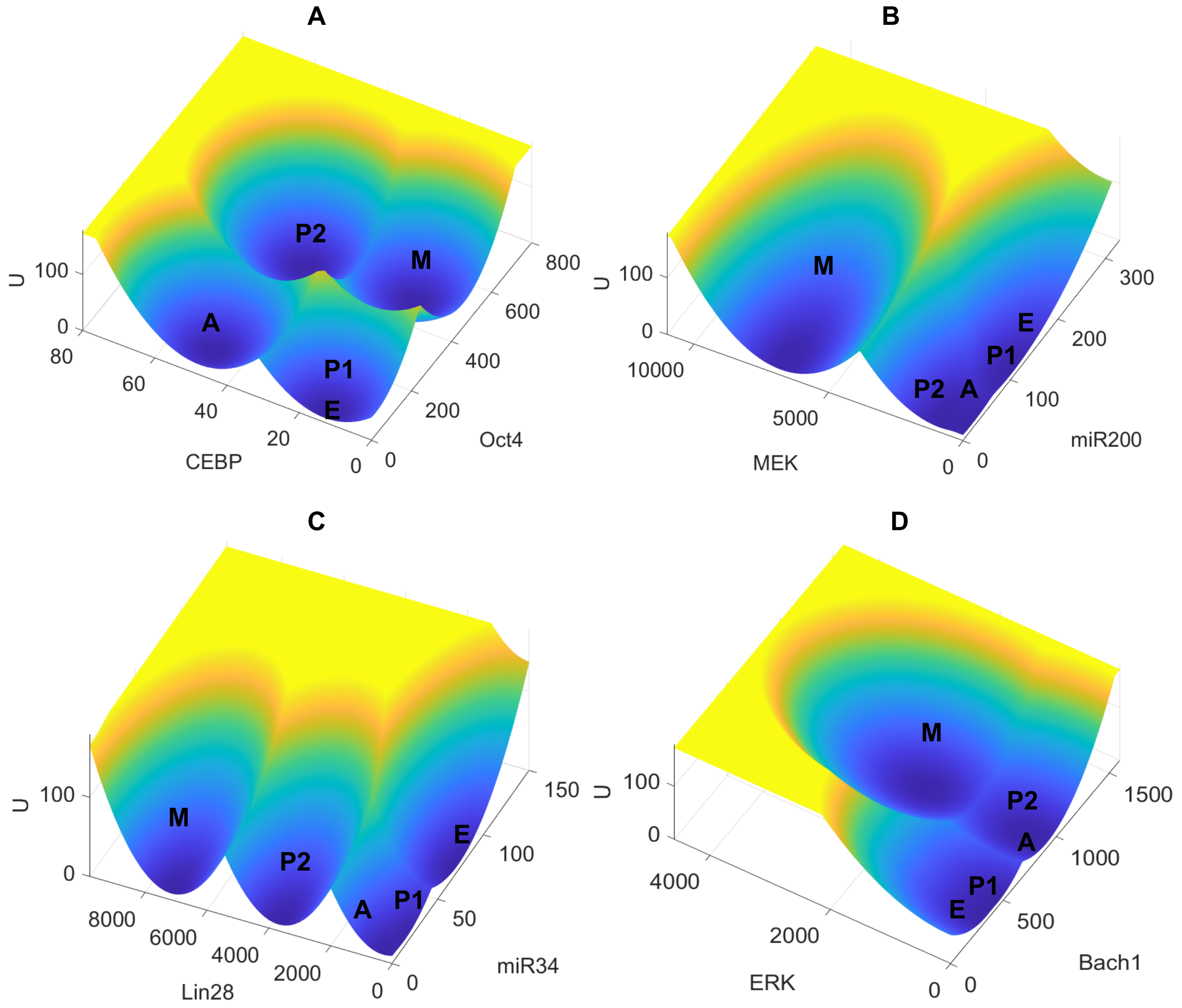


**Figure S6. Exploring the 3-dimensional landscape of the cancer-adipose conversion network using different coordinate pairs.** (A) Landscape shown with CEBP$\alpha$ and OCT4. The CEBP axis represents the expression level of CEBP$\alpha$. The OCT4 axis represents the expression level of OCT4. The U axis represents the potential energy. (B) Landscape shown with MEK and miR200. (C) Landscape shown with LIN28 and miR34. (D) Landscape shown with ERK and BACH1. Stable states include epithelial (E), mesenchymal (M), adipose (A), and intermediate states P1/P2.

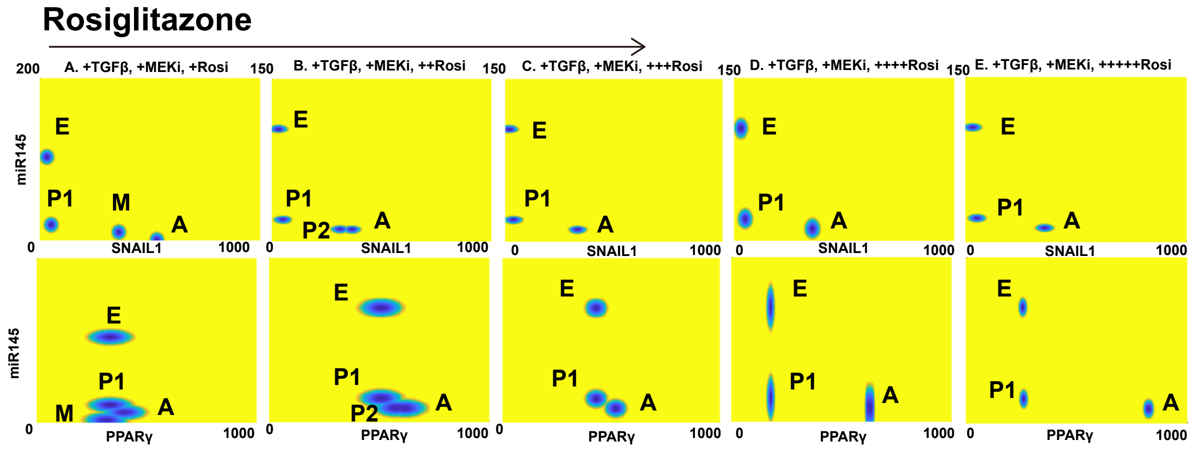


**Figure S7. Rosiglitazone with moderate TGF-$\beta$ and MEK inhibitor promote the transition from M to P2, and P2 to A state.** (A-F) Landscape changes as Rosiglitazone concentration increases, along with moderate TGF-$\beta$ and MEK inhibitor, promoting the transition from mesenchymal (M) to adipose (A) state through partial EMT state P2. (A) Moderate Rosi is added (same as the Figure 2A in main text). (B) Double the dose of Rosi. (C) Three times the dose of Rosi is added. (D) Four times the dose of Rosi is added. (E) Five times the dose of Rosi is added. The attractors are shown with reduced noise for better visualization. The miR145 axis represents the relative concentration of miR145, the SNAI1 axis represents the relative concentration of SNAI1, and the PPAR axis represents the relative concentration of PPAR$\gamma$. The U axis represents the potential energy.

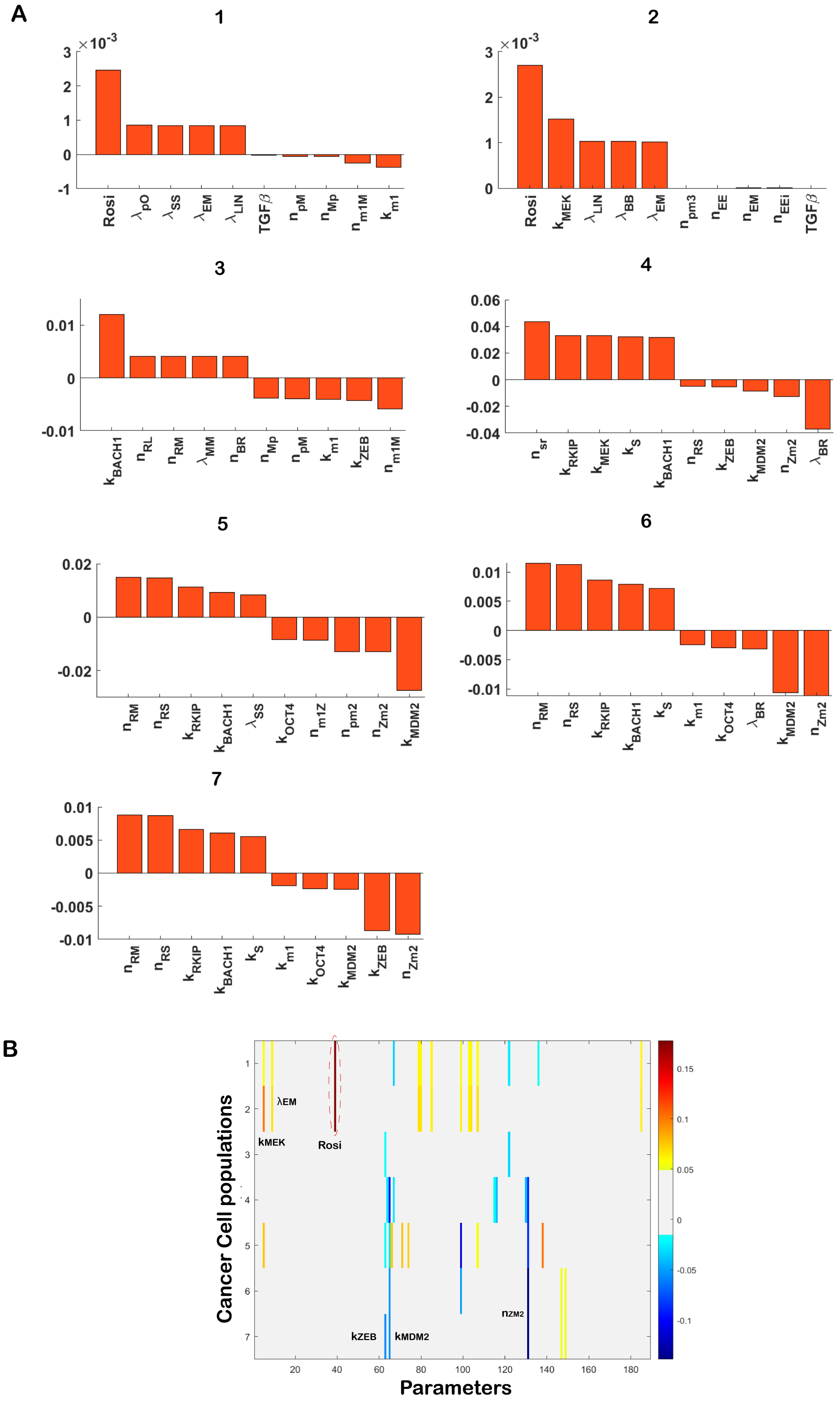


**Figure S8. Robustness of landscape control against heterogeneity in cancer populations.** Random initial parameters to simulate different cancer cell populations. (A) is the top 10 parameters under landscape control to maximize A state (corresponding to Figure 4B in main text). (B) is the result for all 189 parameters under landscape control. The color bars represent the alterations of the parameters. The landscape control results are robust against perturbations in initial parameters (characterizing the heterogeneity in cancer populations).

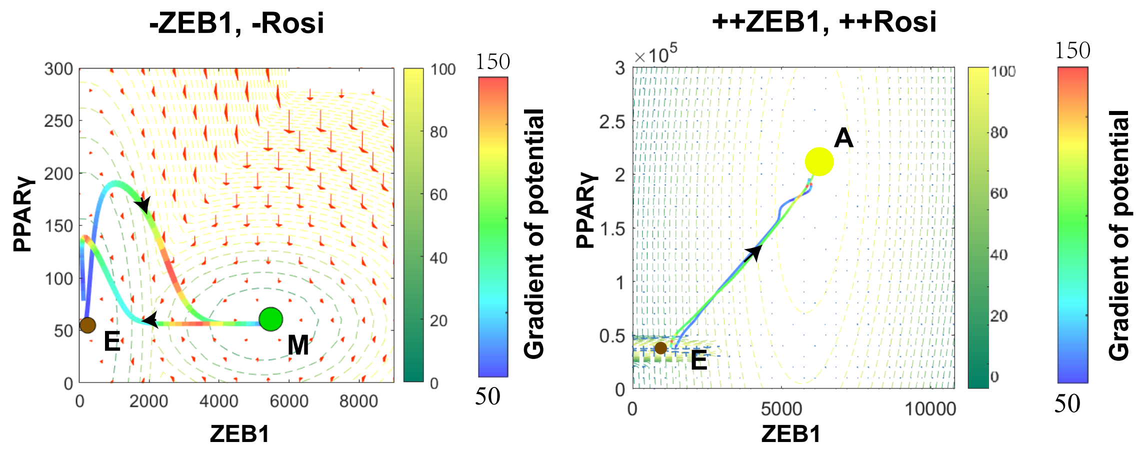


**Figure S9. Effect of ZEB1 and Rosiglitazone on cancer-adipose conversion.** (A) Knockout of ZEB1 and Rosi. (B) Overexpression of ZEB1 and Rosi. The x axis is the expression level of ZEB1, and the y axis is the expression level of PPAR$\gamma$. The green, yellow, and brown balls represent M, A, and E states, respectively, with the size indicating the occupancy of the corresponding states. The potential gradient indicates the transition difficulty between states. Results demonstrate that overexpressing ZEB1 and adding Rosiglitazone promotes cancer cell transition to the A state.

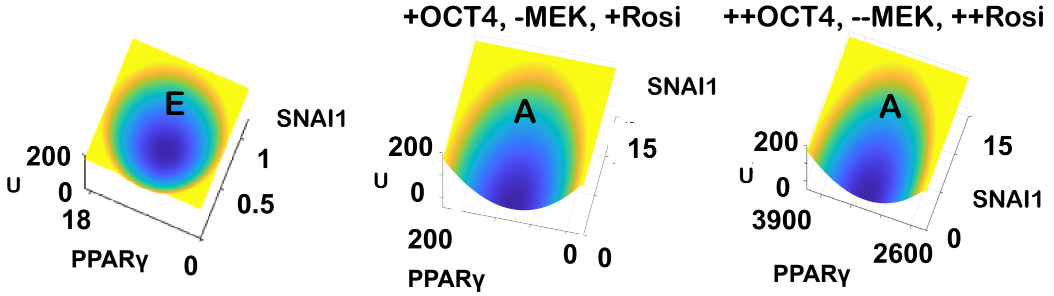


**Figure S10. Using landscape control method to predict drug combinations.** The drug combinations including OCT4, MEK and Rosi, which we select based on the results in Figure S8 (we select Rosi and MEK based on experiment 1-2, and select OCT4 based on experiment 5-7).

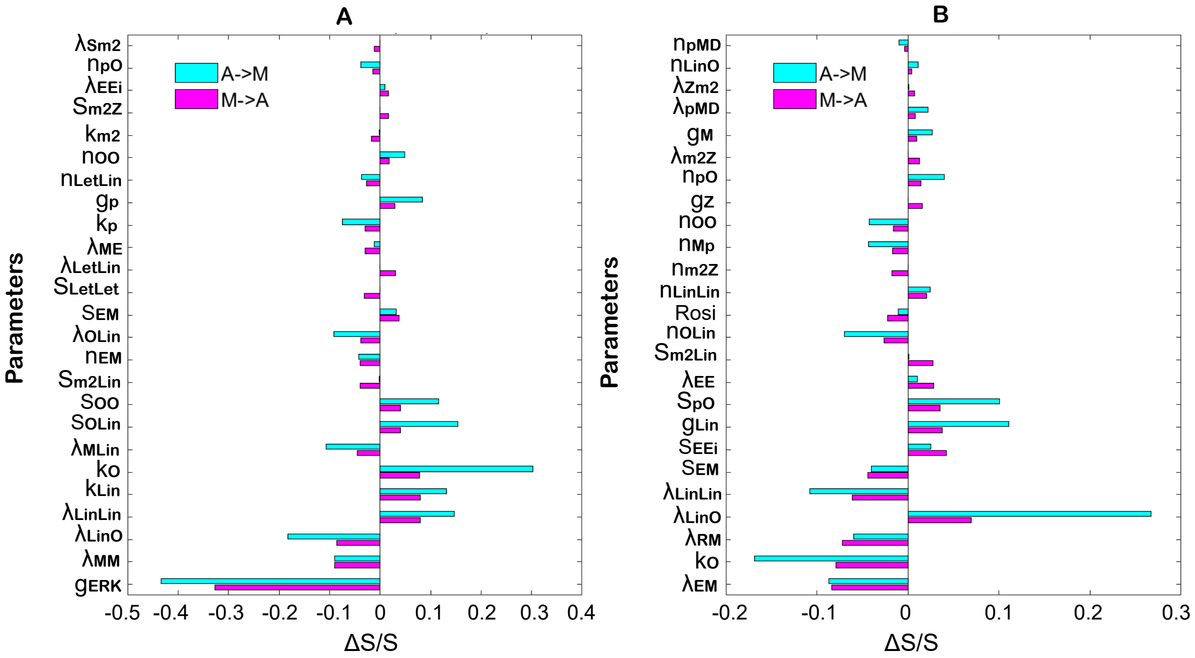


**Figure S11. Sensitivity analysis for the 20 most critical parameters controlling the transition between A and M.** The x axis represents the relative change of transition action ($S$) compared with the unperturbed system, denoted by $\triangle$S/S. The y axis represents the perturbed parameters. Here, each parameter increases or decreases by 10%. Magenta bar represents the A to M transition, and cyan bar represents the M to A transition. (A) Each parameter 10% increases in the transition between A and M. (B) Each parameter 10% decreases in the transition between A and M.

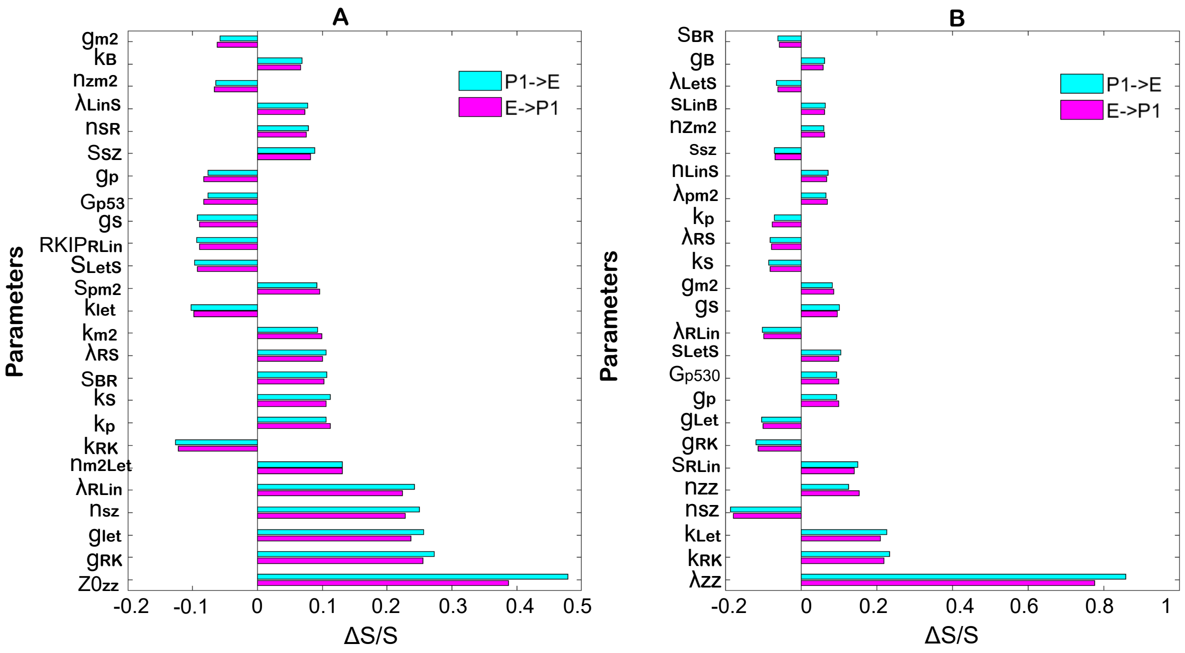


**Figure S12. Sensitivity analysis for the 20 most critical parameters controlling the transition between E and P1.** The x axis represents the relative change of transition action ($S$) compared with the unperturbed system, denoted by △S/S. Y represents the parameters being perturbed. Each parameter is increased or decreased by 10%. Magenta bar represents the P1 to E transition, and cyan bar represents the E to P1 transition. (A) Each parameter 10% increases in the transition between E and P1. (B) Each parameter 10% decreases in the transition between E and P1.

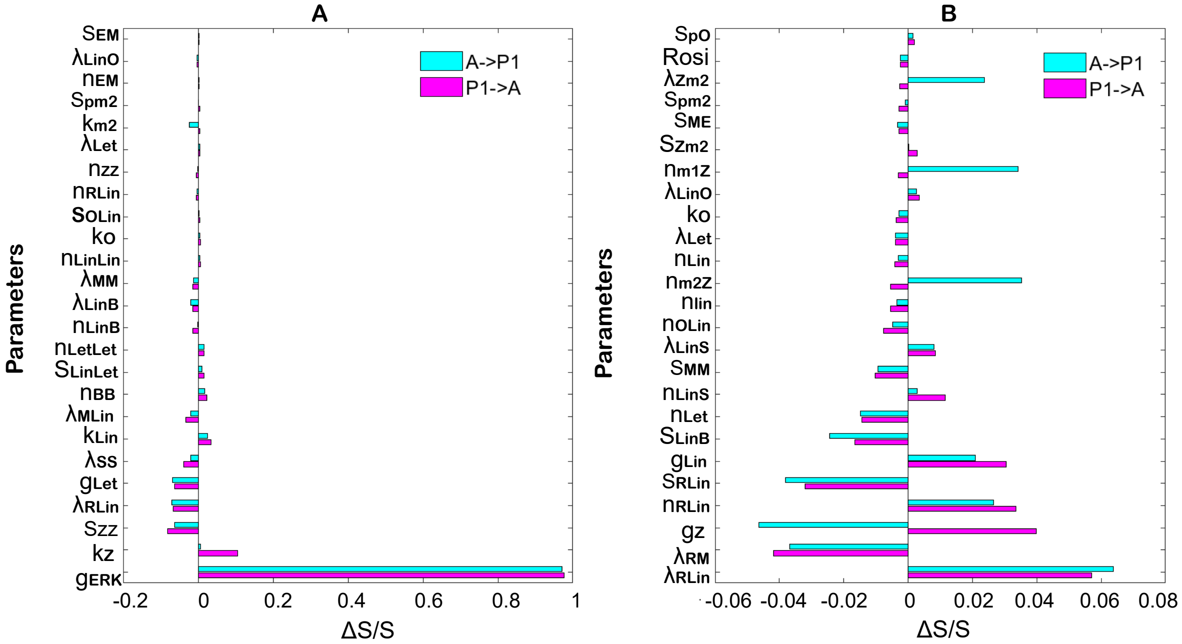


**Figure S13. Sensitivity analysis for the 20 most critical parameters controlling the transition between A and P1.** The x axis represents the relative change of transition action ($S$) compared with the unperturbed system, denoted by $\triangle$S/S. Each parameter is increased or decreased by 10%. Y represents the parameters being perturbed. Magenta bar represents the P1 to A transition, and cyan bar represents the A to P1 transition. (A) Each parameter 10% increases in the transition between A and P1. (B) Each parameter 10% decreases in the transition between A and P1.

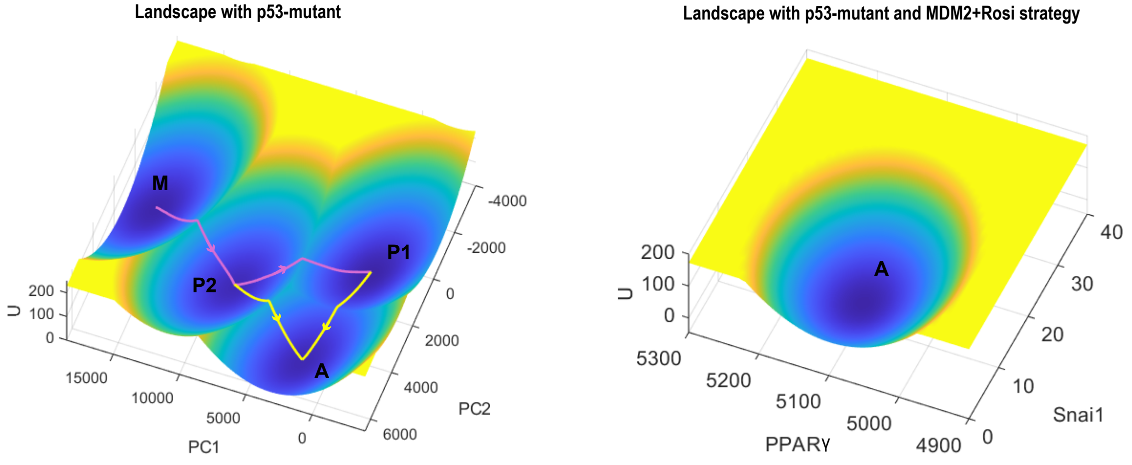


**Figure S14. The effect of rosiglitazone and overexpressing MDM2 strategy in the p53-mutant cells.** (A) Landscape of the p53-mutant cells. The red trajectories represent the transition from M state to P2 state, and from P2 state to P1 state. The yellow trajectories represent the transition from P2 state to A state, and the transition from P1 state to A state. PC1 and PC2 represent the top two principle components from the dimension reduction of landscape. (B) Landscape for overexpression of MDM2 and addition of rosiglitazone in p53-mutant cells. Snail1 is the marker of M state, and PPAR$\gamma$ is the marker of the A state.

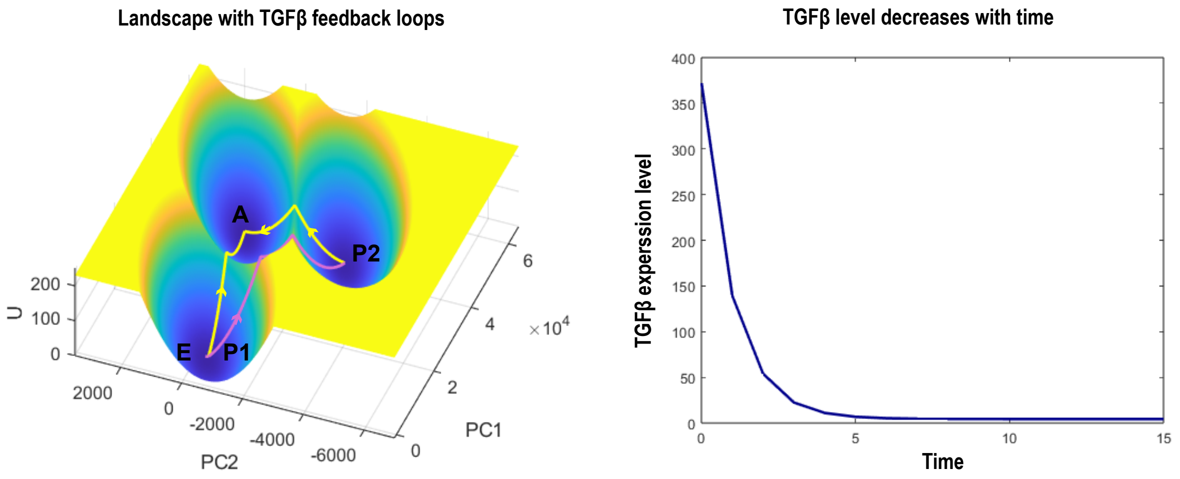


**Figure S15. Landscape with the TGF-$\beta$ negative feedback loop.** (A) Landscape of the model with TGF$\beta$ feedback loops. The other parameters are the same as the model in figure 2 of main text. We can see E, P1, P2 and A state on the landscape with the negative feedback of TGF-$\beta$. (B) With the TGF-$\beta$ negative feedback loop, the expression level of TGF-$\beta$ decreases with time.

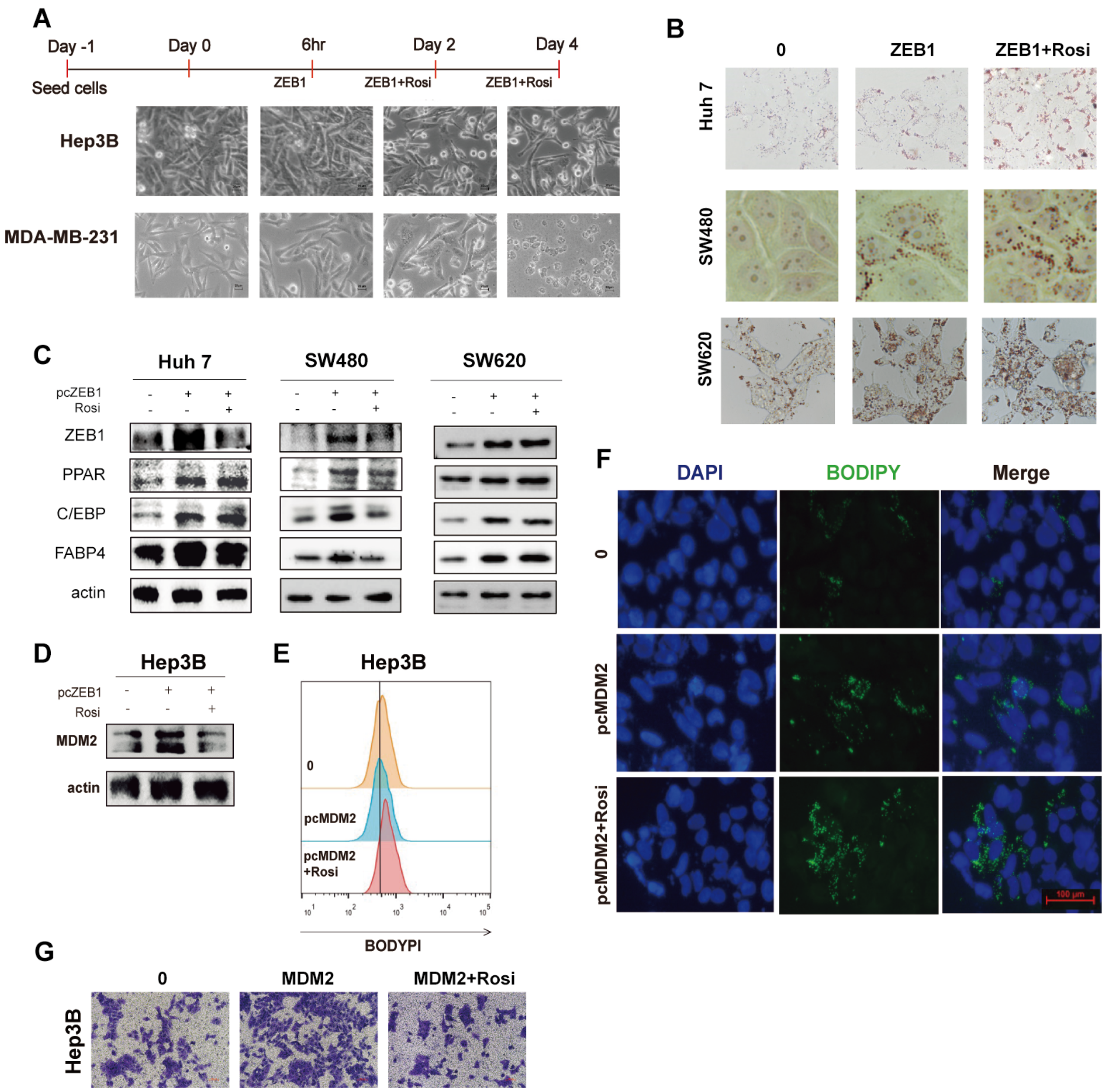


**Figure S16. The combination of rosiglitazone and overexpressing ZEB1 or MDM2 promotes the transformation of tumor cells into adipose cells.** (A) Morphological changes of Hep3B and MDA-MB-231 cells exposed to ZEB1 activator and Rosiglitazone for the time indicated (magnification, x100). (B) Oil red staining performed on the Huh-7, SW480 and SW620 to detect intracellular lipid droplets (magnification, x100). (C) The expression of adipocyte-related proteins was verified by western blot. (D) MDM2 protein expression was elevated when ZEB1 was overexpressed. (E) Flow cytometry was used to detect the changes of BODIPY after MDM2 combined with rosiglitazone. (F) After 24h of rosiglitazone treatment, lipid droplets and nuclei were visualized by immunofluorescence staining with BODIPY (Green) and DAPI (blue). Scale bars, 100 mm. (G) Transwell assay was used to detect the migration capacity of Hep3B treated with MDM2 and rosiglitazone.

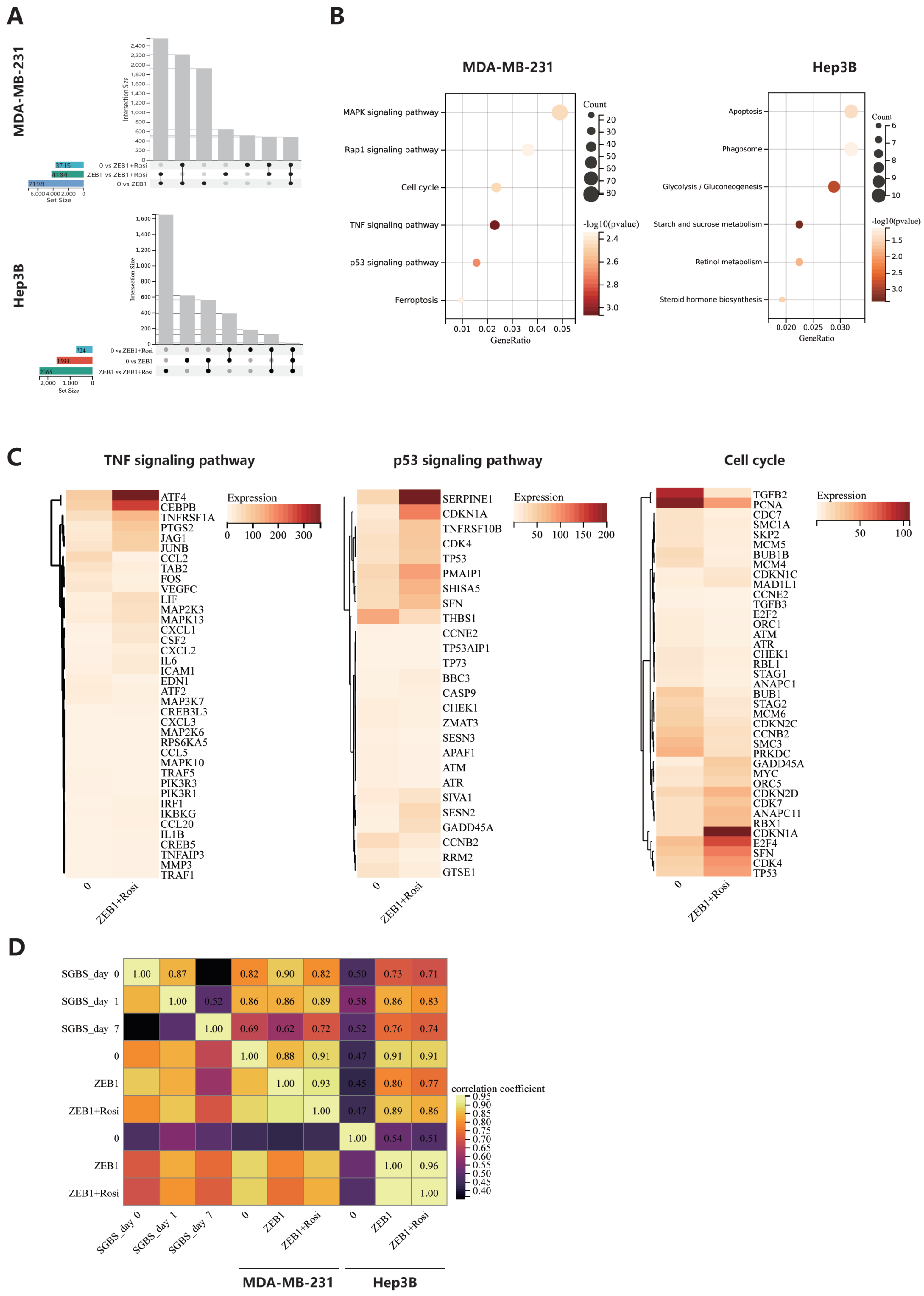


**Figure S17. RNA-seq data analyses support that predicted combination drugs are effective for inducing CAC.** (A) Venn diagram shows the number of differential genes at different intersections for two cell lines MDA-MB-231 and Hep3B. (B) KEGG pathway enrichment analysis of differential genes ($FDR < 0.1$, $p < 0.05$), for cell line MDA-MB-231 and Hep3B. (C) Heat maps show comparisons of gene expression in different groups of MDA-MB-231 cell lines for the enrichment pathway. (D) Correlation analysis of gene expression between cells with combination drug treatment (MDA-MB-231 and Hep3B cell line) and SGBS cells (human adipocyte cells) at different time points of differentiation (GSE161111, 6728 genes shared by the two datasets are used for correlation analysis).

**Table S1.** Experimental evidences of the regulations in CAC network. Here, ”1” represent activation, ”-1” represent inhibition. The CAC network includes total 52 regulations.

| Source Node | Target Node | Interaction Type | References |
|---|---|---|---|
| SNAI1 | ZEB1 | 1 | [28] |
| ZEB1 | ZEB1 | 1 | [28] |
| miR200 | ZEB1 | -1 | [28] |
| miR145 | ZEB1 | -1 | [28] |
| P53 | OCT4 | 1 | [9, 14] |
| OCT4 | OCT4 | 1 | [28] |
| LIN28 | OCT4 | 1 | [1] |
| miR145 | OCT4 | -1 | [9, 14] |
| P53 | MDM2 | 1 | [24] |
| miR145 | MDM2 | -1 | [28] |
| SNAI1 | SNAI1 | -1 | [28] |
| miR34 | SNAI1 | -1 | [28] |
| Let7 | SNAI1 | -1 | [28] |
| OCT4 | miR145 | -1 | [25] |
| ZEB1 | miR145 | -1 | [17] |
| P53 | miR145 | 1 | [10] |
| OCT4 | miR200 | 1 | [25] |
| P53 | miR200 | 1 | [10] |
| ZEB1 | miR200 | -1 | [10] |
| SNAI1 | miR200 | -1 | [28] |
| SNAI1 | miR34 | -1 | [28] |
| ZEB1 | miR34 | -1 | [10] |
| P53 | miR34 | 1 | [10] |
| Mdm2 | P53 | 1 | [24] |
| BACH1 | RKIP | 1 | [2, 12] |
| SNAI1 | RKIP | -1 | [28] |
| RKIP | Let7 | 1 | [2, 12] |
| RKIP | LIN28 | -1 | [26] |
| miR200 | LIN28 | -1 | [25] |
| LIN28 | Let7 | -1 | [28] |
| Let7 | Let7 | 1 | [28] |
| LIN28 | LIN28 | 1 | [1] |
| Let7 | LIN28 | -1 | [28] |
| miR200 | Let7 | 1 | [25] |
| BACH1 | BACH1 | -1 | [12] |
| Let7 | BACH1 | -1 | [3] |
| RKIP | MEK | -1 | [22] |
| RKIP | SNAI1 | -1 | [26] |
| RKIP | LIN28 | -1 | [26] |
| MEK | MEK | 1 | [27] |
| MEK | LIN28 | 1 | [1] |
| OCT4 | LIN28 | 1 | [1, 25] |
| ERK | MEK | -1 | [27] |
| MEK | ERK | 1 | [17, 18] |
| ERK | ERK | 1 | [11] |
| ERK | ERK | -1 | [23] |
| MEK | CEBP$\alpha$ | 1 | [6, 7] |
| PPAR$\gamma$ | CEBP$\alpha$ | 1 | [6, 7] |
| ERK | CEBP$\alpha$ | -1 | [6, 7] |
| MEK | PPAR$\gamma$ | 1 | [6, 7] |
| CEBP$\alpha$ | PPAR$\gamma$ | 1 | [6, 7] |
| ERK | PPAR$\gamma$ | 1 | [6, 7] |

**Table S2.** Unified parameter values. Five sets of typical parameters from parameter searching are shown. The searching range for different parameters are: a (0, 20), d (0, 2), $\lambda_a$ (1, 20), $\lambda_i$ (0, 1), n (2, 6), L (0, 1000), TGF-$\beta$ (1, 10), MEKi (0, 1.2), and Rosi (0, 30). More detailed parameter values are shown in Table S3.

| Params index | a | n | d | L | $\lambda_a$ | $\lambda_i$ | TGF-$\beta$ | MEKi | Rosi | Num of stable states |
|---|---|---|---|---|---|---|---|---|---|---|
| 1 | 10 | 2 | 0.3 | 800 | 10 | 0.2 | 4.3 | 0.02 | 1.5 | 4 |
| 2 | 20 | 6 | 0.7 | 700 | 19 | 0.3 | 5.0 | 0.14 | 11.0 | 3 |
| 3 | 19 | 7 | 0.8 | 800 | 18 | 0.4 | 5.0 | 0.14 | 11.0 | 3 |
| 4 | 15 | 3 | 0.9 | 400 | 14 | 0.1 | 3.8 | 0.27 | 27.0 | 4 |
| 5 | 14 | 5 | 1.7 | 200 | 13 | 0.2 | 3.5 | 0.80 | 11.0 | 4 |

**Table S3.** Parameter descriptions and local sensitivity analysis of the five stable states model. The parameters of five stable states model are tuned from unified parameters 1 in Table S2. The "Local sensitivity" column shows the range of parameter perturbations in percentage with the unchanged stable state number. For example, "-99.9" means this parameter can be decreased 99.9% without changing the number of stable states, and ">100" means this value can be increased more than 100% without changing the number of stable states.

| Parameter | Description | Unified parameters | Value | Unit | Local sensitivity (%) |
|---|---|---|---|---|---|
| $g_Z$ | Synthesis rate of ZEB | a | 10 | 1/min | -4.5-8.7 |
| $g_O$ | Synthesis rate of OCT4 | a | 10 | 1/min | -14.4-38.4 |
| $g_{MD}$ | Synthesis rate of Mdm2 | a | 10 | 1/min | -99.9->100 |
| $g_S$ | Synthesis rate of SNAIL1 | a | 10 | 1/min | -5.6-0.4 |
| $g_{m1}$ | Synthesis rate of miR145 | a | 10 | 1/min | -99.9-86.0 |
| $g_{m2}$ | Synthesis rate of miR200 | a | 10 | 1/min | -99.9-77.5 |
| $g_{m3}$ | Synthesis rate of miR34 | a | 10 | 1/min | -99.9->100 |
| $g_p$ | Synthesis rate of P53 | a | 10 | 1/min | -40.7-49.8 |
| $g_{Let}$ | Synthesis rate of Let7 | a | 10 | 1/min | -22.7-21.9 |
| $g_{Lin}$ | Synthesis rate of LIN28 | a | 10 | 1/min | -18.0-16.0 |
| $g_{MEK}$ | Synthesis rate of MEK | a | 10 | 1/min | -15.6-1.1 |
| $g_{ERK}$ | Synthesis rate of ERK | a | 10 | 1/min | -68.8-1.4 |
| $g_{CEBP}$ | Synthesis rate of CEBP$\alpha$ | a | 10 | 1/min | -99.9-100 |
| $g_{PPAR}$ | Synthesis rate of PPAR$\gamma$ | a | 10 | 1/min | -99.9-100 |
| $G_{p53}$ | Maximum production rate of P53 | a | 20 | 1/min | -40.7-49.8 |
| $g_{RK}$ | Synthesis rate of RKIP | a | 90 | 1/min | -0.3-4.5 |
| $g_{BACH1}$ | Synthesis rate of BACH1 | a | 100 | 1/min | -10.3-0.8 |
| $k_Z$ | Degradation rate of ZEB | d | 0.3 | 1/min | -8.3-5.0 |
| $k_O$ | Degradation rate of OCT4 | d | 0.3 | 1/min | -27.7- 16.8 |
| $k_{MD}$ | Degradation rate of Mdm2 | d | 0.3 | 1/min | -68.1->200 |
| $k_{m1}$ | Degradation rate of miR145 | d | 0.3 | 1/min | -46.2->200 |
| $k_{m2}$ | Degradation rate of miR200 | d | 0.3 | 1/min | -43.6->200 |

**Table S3** *(Continued)*

| Parameter | Description | Unified parameters | Value | Unit | Local sensitivity (%) |
|---|---|---|---|---|---|
| $k_{m3}$ | Degradation rate of miR34 | d | 0.3 | 1/min | -99.9->200 |
| $k_p$ | Degradation rate of P53 | d | 0.3 | 1/min | -33.3-68.8 |
| $k_{LetLet}$ | Degradation rate of Let7 | d | 0.3 | 1/min | -18.0-29.4 |
| $k_{LinLin}$ | Degradation rate of LIN28 | d | 0.3 | 1/min | -13.8-22.0 |
| $k_{MEK}$ | Degradation rate of MEK | d | 0.3 | 1/min | -1.1-18.5 |
| $k_{ERK}$ | Degradation rate of ERK | d | 0.3 | 1/min | -1.4->100 |
| $k_{CEBP}$ | Degradation rate of CEBP$\alpha$ | d | 0.3 | 1/min | -99.9->100 |
| $k_{PPAR}$ | Degradation rate of PPAR$\gamma$ | d | 0.3 | 1/min | -99.9->100 |
| $k_{RK}$ | Degradation rate of RKIP | d | 0.06 | 1/min | -4.3-0.4 |
| $k_{BACH1}$ | Degradation rate of BACH1 | d | 0.06 | 1/min | -0.8-11.4 |
| $k_S$ | Degradation rate of SNAIL1 | d | 0.0375 | 1/min | -0.4-5.8 |
| $\lambda_{MM}$ | Fold change of MEK to MEK | $\lambda_a$ | 2 | 1/min | -73.6-3.7 |
| $\lambda_{SZ}$ | Fold change of Snai1 to ZEB | $\lambda_a$ | 10 | 1/min | -63.0->100 |
| $\lambda_{ZZ}$ | Fold change of ZEB to ZEB | $\lambda_a$ | 10 | 1/min | -4.4-21.4 |
| $\lambda_{m2Z}$ | Fold change of miR200 to ZEB | $\lambda_a$ | 10 | 1/min | -99.9->100 |
| $\lambda_{m1Z}$ | Fold change of miR145 to ZEB | $\lambda_a$ | 10 | 1/min | -99.9->100 |
| $\lambda_{m1O}$ | Fold change of miR145 to OCT4 | $\lambda_a$ | 10 | 1/min | -99.9->100 |
| $\lambda_{pMD}$ | Fold change of P53 to Mdm2 | $\lambda_a$ | 10 | 1/min | -99.9->100 |
| $\lambda_{m1MD}$ | Fold change of miR145 to Mdm2 | $\lambda_a$ | 10 | 1/min | -99.9->100 |
| $\lambda_{m3S}$ | Fold change of miR34 to Snai1 | $\lambda_a$ | 10 | 1/min | -99.9->100 |
| $\lambda_{LetS}$ | Fold change of Let7 to Snai1 | $\lambda_a$ | 10 | 1/min | -56.0->100 |
| $\lambda_{Om1}$ | Fold change of OCT4 to miR145 | $\lambda_a$ | 10 | 1/min | -99.9->100 |
| $\lambda_{Zm1}$ | Fold change of ZEB to miR145 | $\lambda_a$ | 10 | 1/min | -63.4->100 |
| $\lambda_{pm1}$ | Fold change of P53 to miR145 | $\lambda_a$ | 10 | 1/min | -99.9->100 |
| $\lambda_{pm2}$ | Fold change of P53 to miR200 | $\lambda_a$ | 10 | 1/min | -99.9->100 |

**Table S3** *(Continued)*

| Parameter | Description | Unified parameters | Value | Unit | Local sensitivity (%) |
|---|---|---|---|---|---|
| $\lambda_{Zm2}$ | Fold change of ZEB to miR200 | $\lambda_a$ | 10 | 1/min | -49.8->100 |
| $\lambda_{Sm2}$ | Fold change of Snai1 to miR200 | $\lambda_a$ | 10 | 1/min | -99.9->100 |
| $\lambda_{Sm3}$ | Fold change of Snai1 to miR34 | $\lambda_a$ | 10 | 1/min | -99.9->100 |
| $\lambda_{Zm3}$ | Fold change of ZEB to miR34 | $\lambda_a$ | 10 | 1/min | -99.9->100 |
| $\lambda_{pm3}$ | Fold change of P53 to miR34 | $\lambda_a$ | 10 | 1/min | -99.9->100 |
| $\lambda_{MDp}$ | Fold change of Mdm2 to P53 | $\lambda_a$ | 10 | 1/min | -99.9->100 |
| $\lambda_{SR}$ | Fold change of Snai1 to RKIP | $\lambda_a$ | 10 | 1/min | -5.8-0.4 |
| $\lambda_{m2Lin}$ | Fold change of miR200 to LIN28 | $\lambda_a$ | 10 | 1/min | -99.9->100 |
| $\lambda_{LinLet}$ | Fold change of LIN28 to Let7 | $\lambda_a$ | 10 | 1/min | -82.1->100 |
| $\lambda_{LetLin}$ | Fold change of Let7 to LIN28 | $\lambda_a$ | 10 | 1/min | -99.9->100 |
| $\lambda_{m2Let}$ | Fold change of miR200 to Let7 | $\lambda_a$ | 10 | 1/min | -99.9->100 |
| $\lambda_{LetB}$ | Fold change of Let7 to BACH1 | $\lambda_a$ | 10 | 1/min | -73.4->100 |
| $\lambda_{RS}$ | Fold change of RKIP to Snai1 | $\lambda_a$ | 10 | 1/min | -1.1-10.8 |
| $\lambda_{OLin}$ | Fold change of OCT4 to LIN28 | $\lambda_a$ | 10 | 1/min | -26.2-84.2 |
| $\lambda_{EE}$ | Fold change of ERK to ERK | $\lambda_a$ | 10 | 1/min | -72.2-6.1 |
| $\lambda_{EEi}$ | Fold change of ERK to ERK | $\lambda_a$ | 10 | 1/min | -99.9->100 |
| $\lambda_{MC}$ | Fold change of MEK to CEBP$\alpha$ | $\lambda_a$ | 10 | 1/min | -99.9->100 |
| $\lambda_{PC}$ | Fold change of PPAR$\gamma$ to CEBP$\alpha$ | $\lambda_a$ | 10 | 1/min | -99.9->100 |
| $\lambda_{EC}$ | Fold change of ERK to CEBP$\alpha$ | $\lambda_a$ | 10 | 1/min | -99.9->100 |
| $\lambda_{MP}$ | Fold change of MEK to PPAR$\gamma$ | $\lambda_a$ | 10 | 1/min | -99.9->100 |
| $\lambda_{CP}$ | Fold change of CEBP$\alpha$ to PPAR$\gamma$ | $\lambda_a$ | 10 | 1/min | -99.9->100 |
| $\lambda_{EP}$ | Fold change of ERK to PPAR$\gamma$ | $\lambda_a$ | 10 | 1/min | -99.9->100 |
| $\lambda_{LinO}$ | Fold change of LIN28 to OCT4 | $\lambda_a$ | 15 | 1/min | -14.6-47.1 |
| $\lambda_{Om2}$ | Fold change of OCT4 to miR200 | $\lambda_a$ | 15 | 1/min | -99.9->100 |
| $\lambda_{RLet}$ | Fold change of RKIP to Let7 | $\lambda_a$ | 15 | 1/min | -23.7-22.5 |

**Table S3** *(Continued)*

| Parameter | Description | Unified parameters | Value | Unit | Local sensitivity (%) |
|---|---|---|---|---|---|
| $\lambda_{MLin}$ | Fold change of MEK to LIN28 | $\lambda_a$ | 15 | 1/min | -18.8-16.8 |
| $\lambda_{ME}$ | Fold change of MEK to ERK | $\lambda_a$ | 24 | 1/min | -69.2-10.5 |
| $\lambda_{RM}$ | Fold change of RKIP to MEK | $\lambda_a$ | 120 | 1/min | -1.2-20.5 |
| $\lambda_{SS}$ | Fold change of Snai1 to Snai1 | $\lambda_i$ | 0.2 | 1/min | -86.5-6.0 |
| $\lambda_{pO}$ | Fold change of P53 to OCT4 | $\lambda_i$ | 0.2 | 1/min | -99.9->100 |
| $\lambda_{OO}$ | Fold change of OCT4 to OCT4 | $\lambda_i$ | 0.2 | 1/min | -99.9->100 |
| $\lambda_{BR}$ | Fold change of BACH1 to RKIP | $\lambda_i$ | 0.2 | 1/min | -1.6-23.2 |
| $\lambda_{EM}$ | Fold change of ERK to MEK | $\lambda_i$ | 0.2 | 1/min | -99.9->100 |
| $\lambda_{LetLet}$ | Fold change of Let7 to Let7 | $\lambda_i$ | 0.2 | 1/min | -99.9->100 |
| $\lambda_{LinLin}$ | Fold change of LIN28 to LIN28 | $\lambda_i$ | 0.2 | 1/min | -99.9-44.0 |
| $\lambda_{BB}$ | Fold change of BACH1 to BACH1 | $\lambda_i$ | 0.2 | 1/min | -58.4-3.8 |
| $\lambda_{RLin}$ | Fold change of RKIP to LIN28 | $\lambda_i$ | 0.2 | 1/min | -99.9->100 |
| $n_{SZ}$ | Hill coefficient for Snai1 activate ZEB | n | 2 | 1/min | -26.6-22.1 |
| $n_{ZZ}$ | Hill coefficient for ZEB activate ZEB | n | 2 | 1/min | -8.5->100 |
| $n_{m2Z}$ | Hill coefficient for miR200 inhibit ZEB | n | 2 | 1/min | -18.7->100 |
| $n_{m1Z}$ | Hill coefficient for miR145 inhibit ZEB | n | 2 | 1/min | -18.5->100 |
| $n_{pO}$ | Hill coefficient for P53 activate OCT4 | n | 2 | 1/min | -74.7->100 |
| $n_{OO}$ | Hill coefficient for OCT4 activate OCT4 | n | 2 | 1/min | -84.3->100 |
| $n_{LinO}$ | Hill coefficient for LIN28 activate OCT4 | n | 2 | 1/min | -46.7->100 |
| $n_{m1O}$ | Hill coefficient for miR145 inhibit OCT4 | n | 2 | 1/min | -59.4->100 |
| $n_{pMD}$ | Hill coefficient for P53 activate Mdm2 | n | 2 | 1/min | -99.9->100 |
| $n_{m1MD}$ | Hill coefficient for miR145 inhibit Mdm2 | n | 2 | 1/min | -99.9->100 |
| $n_{SS}$ | Hill coefficient for Snai1 inhibit Snai1 | n | 2 | 1/min | -24.5-2.7 |
| $n_{m3S}$ | Hill coefficient for miR34 inhibit Snai1 | n | 2 | 1/min | -55.2->100 |
| $n_{LetS}$ | Hill coefficient for Let7 inhibit Snai1 | n | 2 | 1/min | -16.8-83.3 |

**Table S3** *(Continued)*

| Parameter | Description | Unified parameters | Value | Unit | Local sensitivity (%) |
|---|---|---|---|---|---|
| $n_{Om1}$ | Hill coefficient for OCT4 inhibit miR145 | n | 2 | 1/min | -99.9->100 |
| $n_{Zm1}$ | Hill coefficient for ZEB inhibit miR145 | n | 2 | 1/min | -99.9->100 |
| $n_{pm1}$ | Hill coefficient for P53 inhibit miR145 | n | 2 | 1/min | -99.9->100 |
| $n_{Om2}$ | Hill coefficient for OCT4 activate miR200 | n | 2 | 1/min | -59.7->100 |
| $n_{pm2}$ | Hill coefficient for P53 activate miR200 | n | 2 | 1/min | -99.9->100 |
| $n_{Zm2}$ | Hill coefficient for ZEB inhibit miR200 | n | 2 | 1/min | -99.9->100 |
| $n_{Sm2}$ | Hill coefficient for Snai1 inhibit miR200 | n | 2 | 1/min | -99.9->100 |
| $n_{Sm3}$ | Hill coefficient for Snai1 inhibit miR34 | n | 2 | 1/min | -99.9->100 |
| $n_{Zm3}$ | Hill coefficient for ZEB inhibit miR34 | n | 2 | 1/min | -99.9->100 |
| $n_{MDp}$ | Hill coefficient for Mdm2 inhibit P53 | n | 2 | 1/min | -50.2->100 |
| $n_{BR}$ | Hill coefficient for BACH1 inhibit RKIP | n | 2 | 1/min | -49.9-3.6 |
| $n_{SR}$ | Hill coefficient for Snai1 inhibit RKIP | n | 2 | 1/min | -0.8-7.3 |
| $n_{RLet}$ | Hill coefficient for RKIP activate Let7 | n | 2 | 1/min | -25.8->100 |
| $n_{RLin}$ | Hill coefficient for RKIP inhibit LIN28 | n | 2 | 1/min | -64.0->100 |
| $n_{m2Lin}$ | Hill coefficient for miR200 inhibit LIN28 | n | 2 | 1/min | -56.0->100 |
| $n_{LinLet}$ | Hill coefficient for LIN28 inhibit Let7 | n | 2 | 1/min | -58.7-73.2 |
| $n_{LetLet}$ | Hill coefficient for Let7 activate Let7 | n | 2 | 1/min | -88.1->100 |
| $n_{LinLin}$ | Hill coefficient for LIN28 activate LIN28 | n | 2 | 1/min | -34.8->100 |
| $n_{LetLin}$ | Hill coefficient for Let7 inhibit LIN28 | n | 2 | 1/min | -60.5->100 |
| $n_{m2Let}$ | Hill coefficient for miR200 activate Let7 | n | 2 | 1/min | -45.6->100 |
| $n_{BB}$ | Hill coefficient for BACH1 inhibit BACH1 | n | 2 | 1/min | -8.4->100 |
| $n_{LetB}$ | Hill coefficient for Let7 inhibit BACH1 | n | 2 | 1/min | -24.7->100 |
| $n_{RM}$ | Hill coefficient for RKIP inhibit MEK | n | 2 | 1/min | -7.8-0.5 |
| $n_{MM}$ | Hill coefficient for MEK activate MEK | n | 2 | 1/min | -5.8->100 |
| $n_{MLin}$ | Hill coefficient for MEK activate LIN28 | n | 2 | 1/min | -99.9->100 |

**Table S3** *(Continued)*

| Parameter | Description | Unified parameters | Value | Unit | Local sensitivity (%) |
|---|---|---|---|---|---|
| $n_{RS}$ | Hill coefficient for RKIP inhibit Snai1 | n | 2 | 1/min | -6.1-0.5 |
| $n_{OLin}$ | Hill coefficient for OCT4 activate LIN28 | n | 2 | 1/min | -17.5-27.3 |
| $n_{ME}$ | Hill coefficient for MEK activate ERK | n | 2 | 1/min | -24.3->100 |
| $n_{MC}$ | Hill coefficient for MEK activate CEBP$\alpha$ | n | 2 | 1/min | -1.8->100 |
| $n_{PC}$ | Hill coefficient for PPAR$\gamma$ activate CEBP$\alpha$ | n | 2 | 1/min | -99.9->100 |
| $n_{EC}$ | Hill coefficient for ERK inhibit CEBP$\alpha$ | n | 2 | 1/min | -99.9->100 |
| $n_{MP}$ | Hill coefficient for MEK activate PPAR$\gamma$ | n | 2 | 1/min | -99.9->100 |
| $n_{CP}$ | Hill coefficient for CEBP$\alpha$ activate PPAR$\gamma$ | n | 2 | 1/min | -99.9->100 |
| $n_{EP}$ | Hill coefficient for ERK inhibit PPAR$\gamma$ | n | 2 | 1/min | -99.9->100 |
| $n_{pm3}$ | Hill coefficient for P53 activate miR34 | n | 4 | 1/min | -99.9->100 |
| $n_{EM}$ | Hill coefficient for ERK inhibit MEK | n | 4 | 1/min | -11.1->100 |
| $n_{EE}$ | Hill coefficient for ERK activate ERK | n | 4 | 1/min | -1.8->100 |
| $n_{EEi}$ | Hill coefficient for ERK inhibit ERK | n | 4 | 1/min | -79.1->100 |
| $S_{MC}$ | Threshold of MEK activate CEBP$\alpha$ | L | 800 | 1/min | -99.9->100 |
| $S_{MP}$ | Threshold of MEK activate PPAR$\gamma$ | L | 800 | 1/min | -99.9->100 |
| $S_{EC}$ | Threshold of ERK inhibit CEBP$\alpha$ | L | 800 | 1/min | -99.9->100 |
| $S_{EP}$ | Threshold of ERK inhibit PPAR$\gamma$ | L | 800 | 1/min | -99.9->100 |
| $S_{CP}$ | Threshold of CEBP$\alpha$ activate PPAR$\gamma$ | L | 800 | 1/min | -99.9->100 |
| $S_{PC}$ | Threshold of PPAR$\gamma$ activate CEBP$\alpha$ | L | 800 | 1/min | -99.9->100 |
| $S_{EM}$ | Threshold of ERK inhibit MEK | L | 800 | 1/min | -9.1->100 |
| $S_{EE}$ | Threshold of ERK activate ERK | L | 800 | 1/min | -1.6->100 |
| $S_{RM}$ | Threshold of RKIP inhibit MEK | L | 800 | 1/min | -9.6-0.6 |
| $S_{MLin}$ | Threshold of MEK activate LIN28 | L | 800 | 1/min | -19.4-26.3 |
| $S_{RS}$ | Threshold of RKIP inhibit Snai1 | L | 800 | 1/min | -6.2-0.7 |
| $S_{OLin}$ | Threshold of OCT4 activate LIN28 | L | 800 | 1/min | -28.7-22.5 |

**Table S3** *(Continued)*

| Parameter | Description | Unified parameters | Value | Unit | Local sensitivity (%) |
|---|---|---|---|---|---|
| $S_{BR}$ | Threshold of BACH1 inhibit RKIP | L | 800 | 1/min | -0.4-6.6 |
| $S_{BB}$ | Threshold of BACH1 inhibit BACH1 | L | 800 | 1/min | -14.1-1.1 |
| $S_{ZZ}$ | Threshold of ZEB activate ZEB | L | 800 | 1/min | -10.7-2.7 |
| $S_{Zm1}$ | Threshold of ZEB inhibit miR145 | L | 800 | 1/min | -99.9-85.2 |
| $S_{Zm2}$ | Threshold of ZEB inhibit miR200 | L | 800 | 1/min | -99.9-76.9 |
| $S_{Zm3}$ | Threshold of ZEB inhibit miR34 | L | 800 | 1/min | -99.9->100 |
| $S_{OO}$ | Threshold of OCT4 activate OCT4 | L | 800 | 1/min | -78.5->100 |
| $S_{Om1}$ | Threshold of OCT4 inhibit miR145 | L | 800 | 1/min | -99.9->100 |
| $S_{Om2}$ | Threshold of OCT4 activate miR200 | L | 800 | 1/min | -87.4->100 |
| $S_{MDp}$ | Threshold of Mdm2 inhibit P53 | L | 800 | 1/min | -68.1->100 |
| $S_{SZ}$ | Threshold of Snai1 activate ZEB | L | 800 | 1/min | -55.0-83.1 |
| $S_{SS}$ | Threshold of Snai1 inhibit Snai1 | L | 800 | 1/min | -14.6-1.1 |
| $S_{Sm2}$ | Threshold of Snai1 inhibit miR200 | L | 800 | 1/min | -99.9->100 |
| $S_{Sm3}$ | Threshold of Snai1 inhibit miR34 | L | 800 | 1/min | -99.9->100 |
| $S_{SR}$ | Threshold of Snai1 inhibit RKIP | L | 800 | 1/min | -0.3-4.5 |
| $S_{m1Z}$ | Threshold of miR145 inhibit ZEB | L | 800 | 1/min | -46.7->100 |
| $S_{m1O}$ | Threshold of miR145 inhibit OCT4 | L | 800 | 1/min | -99.9->100 |
| $S_{m1MD}$ | Threshold of miR145 inhibit Mdm2 | L | 800 | 1/min | -99.9->100 |
| $S_{m2Z}$ | Threshold of miR200 inhibit ZEB | L | 800 | 1/min | -47.3->100 |
| $S_{m2Let}$ | Threshold of miR200 activate Let7 | L | 800 | 1/min | -78.4->100 |
| $S_{m3S}$ | Threshold of miR34 inhibit Snai1 | L | 800 | 1/min | -99.9->100 |
| $S_{pO}$ | Threshold of P53 activate OCT4 | L | 800 | 1/min | -25.0->100 |
| $S_{pMD}$ | Threshold of P53 activate Mdm2 | L | 800 | 1/min | -75.1->100 |
| $S_{pm1}$ | Threshold of miR200 inhibit ZEB | L | 800 | 1/min | -54.1->100 |
| $S_{pm2}$ | Threshold of P53 activate miR200 | L | 800 | 1/min | -49.8->100 |

**Table S3** *(Continued)*

| Parameter | Description | Unified parameters | Value | Unit | Local sensitivity (%) |
|---|---|---|---|---|---|
| $S_{pm3}$ | Threshold of P53 activate miR34 | L | 800 | 1/min | -99.9->100 |
| $S_{RLet}$ | Threshold of RKIP activate Let7 | L | 800 | 1/min | -25.8-37.7 |
| $S_{LetS}$ | Threshold of Let7 inhibit Snai1 | L | 800 | 1/min | -38.3-79.3 |
| $S_{LetLet}$ | Threshold of Let7 activate Let7 | L | 800 | 1/min | -25.0-100 |
| $S_{LetLin}$ | Threshold of Let7 inhibit LIN28 | L | 800 | 1/min | -83.3-100 |
| $S_{LetB}$ | Threshold of Let7 inhibit BACH1 | L | 800 | 1/min | -49.8-100 |
| $S_{LinO}$ | Threshold of LIN28 activate OCT4 | L | 800 | 1/min | -25.0-58.1 |
| $S_{LinLet}$ | Threshold of LIN28 inhibit Let7 | L | 800 | 1/min | -99.9-100 |
| $S_{LinLin}$ | Threshold of LIN28 activate LIN28 | L | 800 | 1/min | -25.2-35.0 |
| $S_{RLin}$ | Threshold of RKIP inhibit LIN28 | L | 800 | 1/min | -56.4-100 |
| $S_{m2Lin}$ | Threshold of miR200 inhibit LIN28 | L | 800 | 1/min | -99.9->100 |
| $S_{ME}$ | Threshold of MEK activate ERK | L | 1000 | 1/min | -1.5->100 |
| $S_{MM}$ | Threshold of MEK activate MEK | L | 2000 | 1/min | -4.3->100 |
| $S_{EEi}$ | Threshold of ERK inhibit ERK | L | 3000 | 1/min | -63.8->100 |
| TGF-$\beta$ | TGF-$\beta$ | TGF-$\beta$ | 4.3 | 1/min | -2.3-0.3 |
| MEKi | MEK inhibitor | MEKi | 0.02 | 1/min | -1.1-18.5 |
| Rosi | Rosiglitazone | Rosi | 1.5 | 1/min | -1.4->100 |

**Table S4.** Principle components of the landscape after dimension reduction (corresponding to Figure 2A).

| | PC1 | PC2 |
|---|---|---|
| ZEB1 | 0.442523374279039 | -0.853243068659828 |
| OCT4 | 0.038988973886299 | 0.009398091466662 |
| MDM2 | 0.000869499307402 | -0.004563005539273 |
| SNAI1 | 0.044163666714536 | -0.071419716924926 |
| miR145 | -0.007113086306737 | 0.031375681005492 |
| miR200 | -0.006085865252987 | 0.033529274710443 |
| miR34 | -0.004935815297948 | 0.022900506130479 |
| P53 | -0.001308744892050 | 0.006858495746951 |
| RKIP | -0.093112718572964 | 0.172267586171097 |
| Let7 | -0.049030853168444 | 0.114564292219351 |
| LIN28 | 0.635781136082646 | 0.339725739925689 |
| BACH1 | 0.048388901105210 | -0.098783770765966 |
| MEK | 0.593212509634540 | 0.297164124806886 |
| ERK | 0.176220685096988 | 0.085641208118273 |
| CEBP$\alpha$ | 0.000075130417223 | -0.006845960215214 |
| PPAR$\gamma$ | 0.000264665850194 | -0.020628041745185 |

**Table S5.** Landscape control method predicts potential drug combinations for inducing CAC. We list three combinations of drug targets that can promote A state based on the results of our landscape control method. We have verified the two of these drugs by biological experiments in this work. The other drug combinations warrant further experimental verifications.

| Drug 1 | Drug 2 | Drug 3 | Optimized state | Experiment verified |
|---|---|---|---|---|
| ZEB1 activator | Rosiglitazone | None | A state | ✓ |
| MDM2 activator | Rosiglitazone | None | A state | ✓ |
| OCT4 activator | MEK inhibitor | Rosiglitazone | A state | × |

**Table S6.** Gene expression level for the system with five stable states coexisting. E is epithelial state, A is adipose state, M is mesenchymal state, and P1/P2 are partial/intermediate EMT states.

| Genes | E | M | A | P1 | P2 |
|---|---|---|---|---|---|
| ZEB1 | 138.89 | 5262.61 | 5146.09 | 841.33 | 5264.11 |
| OCT4 | 21.30 | 461.14 | 73.14 | 24.75 | 439.32 |
| MDM2 | 115.38 | 128.18 | 128.01 | 127.56 | 128.18 |
| SNAIL1 | 33.68 | 532.25 | 520.50 | 52.83 | 532.22 |
| miR145 | 104.44 | 4.03 | 12.20 | 22.24 | 4.24 |
| miR200 | 104.48 | 15.62 | 3.98 | 21.87 | 14.77 |
| miR34 | 72.11 | 2.34 | 2.40 | 14.54 | 2.34 |
| P53 | 563.35 | 544.09 | 544.36 | 545.05 | 544.10 |
| RKIP | 1291.23 | 218.43 | 244.60 | 1169.37 | 218.45 |
| Let7 | 597.74 | 6.67 | 30.18 | 430.95 | 6.87 |
| LIN28 | 3.34 | 7180.41 | 319.3893 | 5.86 | 3707.34 |
| BACH1 | 345.03 | 912.33 | 905.93 | 445.41 | 912.31 |
| MEK1 | 82.52 | 6780.50 | 864.06 | 87.50 | 927.21 |
| ERK1 | 25.68 | 2018.90 | 267.16 | 26.11 | 308.57 |
| CEBP$\alpha$ | 13.13 | 13.66 | 47.87 | 13.28 | 42.65 |
| PPAR$\gamma$ | 54.37 | 56.40 | 158.53 | 54.93 | 145.83 |

# References

1. Surabhi Dangi-Garimella, Jieun Yun, Eva M. Eves, Martin Newman, Stefan J. Erkeland, Scott M. Hammond, Andy J. Minn, and Marsha Rich Rosner. Raf kinase inhibitory protein suppresses a metastasis signalling cascade involving LIN28 and let-7. *EMBO Journal*, 28(4):347–358, 2009.

2. Sadaf Davudian, Behzad Mansoori, Neda Shajari, Ali Mohammadi, and Behzad Baradaran. Bach1, the master regulator gene: A novel candidate target for cancer therapy. *Gene*, 588(1):30–37, 2016.

3. Sadaf Davudian, Neda Shajari, Tohid Kazemi, Behzad Mansoori, Shima Salehi, Ali Mohammadi, Dariush Shanehbandi, Vahid Khaze Shahgoli, Milad Asadi, and Behzad Baradaran. Bach1 silencing by sirna inhibits migration of ht-29 colon cancer cells through reduction of metastasis-related genes. *Biomedicine & Pharmacotherapy*, 84:191–198, 2016.

4. Carine Gubelmann, Petra C Schwalie, Sunil K Raghav, Eva Röder, Tenagne Delessa, Elke Kiehlmann, Sebastian M Waszak, Andrea Corsinotti, Gilles Udin, Wiebke Holcombe, et al. Identification of the transcription factor zeb1 as a central component of the adipogenic gene regulatory network. *elife*, 3:e03346, 2014.

5. Bin Huang, Mingyang Lu, Dongya Jia, Eshel Ben-Jacob, Herbert Levine, and Jose N Onuchic. Interrogating the topological robustness of gene regulatory circuits by randomization. *PLoS computational biology*, 13(3):e1005456, 2017.

6. Dana Ishay-Ronen and Gerhard Christofori. Targeting cancer cell metastasis by converting cancer cells into fat. *Cancer research*, 79(21):5471–5475, 2019.

7. Dana Ishay-Ronen, Maren Diepenbruck, Ravi Kiran Reddy Kalathur, Nami Sugiyama, Stefanie Tiede, Robert Ivanek, Glenn Bantug, Marco Francesco Morini, Junrong Wang, Christoph Hess, et al. Gain Fat—Lose Metastasis: Converting Invasive Breast Cancer Cells into Adipocytes Inhibits Cancer Metastasis. *Cancer Cell*, 35(1):17–32.e6, 2019.

8. Xin Kang, Jin Wang, and Chunhe Li. Exposing the Underlying Relationship of Cancer Metastasis to Metabolism and Epithelial-Mesenchymal Transitions. *iScience*, 21:754–772, 2019.

9. Teruhisa Kawamura, Jotaro Suzuki, Yunyuan V Wang, Sergio Menendez, Laura Batlle Morera, Angel Raya, Geoffrey M Wahl, and Juan Carlos Izpisúa Belmonte. Linking the p53 tumour suppressor pathway to somatic cell reprogramming. *Nature*, 460(7259):1140–1144, 2009.

10. Taewan Kim, Angelo Veronese, Flavia Pichiorri, Tae Jin Lee, Young-Jun Jeon, Stefano Volinia, Pascal Pineau, Agnès Marchio, Jeff Palatini, Sung-Suk Suh, et al. p53 regulates epithelial–mesenchymal

transition through micrornas targeting zeb1 and zeb2. *Journal of Experimental Medicine*, 208(5):875–883, 2011.

11. Conan G Kinsey, Soledad A Camolotto, Amelie M Boespflug, Katrin P Guillen, Mona Foth, Amanda Truong, Sophia S Schuman, Jill E Shea, Michael T Seipp, Jeffrey T Yap, et al. Protective autophagy elicited by raf→ mek→ erk inhibition suggests a treatment strategy for ras-driven cancers. *Nature medicine*, 25(4):620–627, 2019.

12. Jiyoung Lee, Jinho Lee, Kevin S Farquhar, Jieun Yun, Casey A Frankenberger, Elena Bevilacqua, Kam Yeung, Eun-Jin Kim, Gábor Balázsi, and Marsha Rich Rosner. Network of mutually repressive metastasis regulators can promote cell heterogeneity and metastatic transitions. *Proceedings of the National Academy of Sciences*, 111(3):E364–E373, 2014.

13. Chunhe Li and Gabor Balazsi. A landscape view on the interplay between emt and cancer metastasis. *npj Syst Biol Appl*, 4(34), 2018.

14. Chunfang Liu, Zhen Cai, Guoxiang Jin, Danni Peng, Bo-Syong Pan, Xian Zhang, Fei Han, Xiaohong Xu, and Hui-Kuan Lin. Abnormal gametogenesis induced by p53 deficiency promotes tumor progression and drug resistance. *Cell discovery*, 4(1):1–16, 2018.

15. Mingyang Lu, Mohit Kumar Jolly, Herbert Levine, José N Onuchic, and Eshel Ben-Jacob. Microrna-based regulation of epithelial–hybrid–mesenchymal fate determination. *Proceedings of the National Academy of Sciences*, 110(45):18144–18149, 2013.

16. Gretel Nusspaumer, Sumit Jaiswal, Andrea Barbero, Robert Reinhardt, Dana Ishay Ronen, Alexander Haumer, Thomas Lufkin, Ivan Martin, and Rolf Zeller. Ontogenic identification and analysis of mesenchymal stromal cell populations during mouse limb and long bone development. *Stem cell reports*, 9(4):1124–1138, 2017.

17. Naoki Oshimori, Daniel Oristian, and Elaine Fuchs. Tgf-$\beta$ promotes heterogeneity and drug resistance in squamous cell carcinoma. *Cell*, 160(5):963–976, 2015.

18. Ievgenia Pastushenko, Audrey Brisebarre, Alejandro Sifrim, Marco Fioramonti, Tatiana Revenco, Soufiane Boumahdi, Alexandra Van Keymeulen, Daniel Brown, Virginie Moers, Sophie Lemaire, et al. Identification of the tumour transition states occurring during emt. *Nature*, 556(7702):463–468, 2018.

19. Deepanwita Prusty, Bae-Hang Park, Kathryn E Davis, and Stephen R Farmer. Activation of mek/erk signaling promotes adipogenesis by enhancing peroxisome proliferator-activated receptor $\gamma$ (ppar$\gamma$) and c/ebp$\alpha$ gene expression during the differentiation of 3t3-l1 preadipocytes. *Journal of Biological Chemistry*, 277(48):46226–46232, 2002.

20. Hanna S Radomska, Daniela S Bassères, Rui Zheng, Pu Zhang, Tajhal Dayaram, Yukiya Yamamoto, David W Sternberg, Nathalie Lokker, Neill A Giese, Stefan K Bohlander, et al. Block of c/ebp$\alpha$ function by phosphorylation in acute myeloid leukemia with flt3 activating mutations. *The Journal of experimental medicine*, 203(2):371–381, 2006.

21. Sascha Sauer. Ligands for the nuclear peroxisome proliferator-activated receptor gamma. *Trends in Pharmacological Sciences*, 36(10):688–704, 2015.

22. Anna Shvartsur, Kevin B Givechian, Hermes Garban, and Benjamin Bonavida. Overexpression of rkip and its cross-talk with several regulatory gene products in multiple myeloma. *Journal of Experimental & Clinical Cancer Research*, 36(1):1–14, 2017.

23. Evrim B Ünal, Florian Uhlitz, and Nils Blüthgen. A compendium of erk targets. *FEBS letters*, 591(17):2607–2615, 2017.

24. Lyubomir T Vassilev, Binh T Vu, Bradford Graves, Daisy Carvajal, Frank Podlaski, Zoran Filipovic, Norman Kong, Ursula Kammlott, Christine Lukacs, Christian Klein, et al. In vivo activation of the p53 pathway by small-molecule antagonists of mdm2. *Science*, 303(5659):844–848, 2004.

25. Guiying Wang, Xudong Guo, Wujun Hong, Qidong Liu, Tingyi Wei, Chenqi Lu, Longfei Gao, Dan Ye, Yi Zhou, Jie Chen, et al. Critical regulation of mir-200/zeb2 pathway in oct4/sox2-induced mesenchymal-to-epithelial transition and induced pluripotent stem cell generation. *Proceedings of the National Academy of Sciences*, 110(8):2858–2863, 2013.

26. Jieun Yun, Casey A Frankenberger, Wen-Liang Kuo, Mirjam C Boelens, Eva M Eves, Nancy Cheng, Han Liang, Wen-Hsiung Li, Hemant Ishwaran, Andy J Minn, et al. Signalling pathway for rkip and let-7 regulates and predicts metastatic breast cancer. *The EMBO journal*, 30(21):4500–4514, 2011.

27. Sven Zimmermann, Christian Rommel, Algirdas Ziogas, Josip Lovric, Karin Moelling, and Gerald Radziwill. Mek1 mediates a positive feedback on raf-1 activity independently of ras and src. *Oncogene*, 15(13):1503–1511, 1997.

28. Dimitrios G Zisoulis, Zoya S Kai, Roger K Chang, and Amy E Pasquinelli. Autoregulation of microrna biogenesis by let-7 and argonaute. *Nature*, 486(7404):541–544, 2012.

29. Y Zuo, L Qiang, and S R Farmer. Activation of CCAAT/enhancer-binding protein (C/EBP) alpha expression by C/EBP beta during adipogenesis requires a peroxisome proliferator-activated receptor-gamma-associated repression of HDAC1 at the C/ebp alpha gene promoter. *J Biol Chem*, 281(12):7960–7967, 2006.
